# Impact of prolonged exposure to occasional and regular waterpipe smoke on cardiac injury, oxidative stress and mitochondrial dysfunction in male mice

**DOI:** 10.3389/fphys.2024.1286366

**Published:** 2024-02-02

**Authors:** Naserddine Hamadi, Suhail Al-Salam, Sumaya Beegam, Nur Elena Zaaba, Ozaz Elzaki, Abderrahim Nemmar

**Affiliations:** ^1^ Department of Life and Environmental Sciences, College of Natural and Health Sciences, Zayed University, Abu Dhabi, United Arab Emirates; ^2^ Department of Pathology, College of Medicine and Health Sciences, United Arab Emirates University, Al Ain, United Arab Emirates; ^3^ Zayed Center for Health Sciences, United Arab Emirates University, Al Ain, United Arab Emirates; ^4^ Department of Physiology, College of Medicine and Health Sciences, United Arab Emirates University, Al Ain, United Arab Emirates

**Keywords:** waterpipe smoke, occasional, regular, cardiac damage, inflammation

## Abstract

Regular waterpipe smoking (Reg-WPS) is well recognized for its deleterious effect on the heart. However, there is a paucity of experimental studies on the impact of occasional waterpipe smoking (Occ-WPS), also known as nondaily smoking, versus Reg-WPS on cardiac homeostasis, and the mechanisms underlying these effects. Hence, we aimed, in the present study, to investigate the effect of Occ-WPS (30 min/day, 1 day/week) versus Reg-WPS (30 min/day, 5 days/week) for 6 months on systolic blood pressure (SBP), cardiac injury, oxidative markers, chemokines, proinflammatory cytokines, DNA damage and mitochondrial function compared with air (control) exposed mice. Our results show that SBP was increased following exposure to either Occ-WPS or Reg-WPS compared with air-exposed mice. Moreover, we found that only Reg-WPS induced a significant elevation in the levels of troponin I, brain natriuretic peptide, lactate dehydrogenase, and creatine phosphokinase. However, the atrial natriuretic peptide (ANP) was significantly increased in both Occ-WPS and Reg-WPS groups. Compared with air-exposed mice, the levels of lipid peroxidation, reduced glutathione and monocyte chemoattractant protein-1 were only significantly augmented in the Reg-WPS. However, catalase, superoxide dismutase, and CXCL1 were significantly increased in both Occ-WPS and Reg-WPS. The concentrations of the adhesion molecules E-selectin, vascular cell adhesion molecule-1, and intercellular adhesion molecule-1 were solely elevated in the heart of mice exposed to Reg-WPS. Similarly, the concentrations of interleukin-1β and tumor necrosis factor α were only significantly augmented in the Reg-WPS. However, both Occ-WPS and Reg-WPS triggered significant augmentation in the levels of IL17 and DNA damage compared to the control groups. Furthermore, while Occ-WPS induced a slight but statistically insignificant elevation in the concentrations of mammalian targets of rapamycin and nuclear factor erythroid-derived 2-like 2 (Nrf2) expression, Reg-WPS exposure increased their levels substantially, in addition to p53 and mitochondrial complexes II & III, and IV activities compared with air-exposed mice. In conclusion, our findings show that while the long-term Occ-WPS exposure induced an elevation of SBP, ANP, antioxidant enzymes, IL17, CXCL1, and cardiac DNA damage, Reg-WPS exposure was consistently associated with the elevation of SBP and occurrence of cardiac damage, inflammation, oxidative stress, DNA damage and mitochondrial dysfunction.

## 1 Introduction

Cardiovascular disease is the leading cause of death worldwide (Mc Namara et al., 2019). Tobacco smoking remains a significant contributor to the development of cardiovascular disease and stands as the primary preventable cause of mortality (Kondo et al., 2019). Cigarette smoking (CS) has declined in recent years in Western countries. In contrast, waterpipe smoking (WPS) has increased, especially among youth (Jukema et al., 2014). Due to the rise in the number of WPS users, the World Health Organization listed WPS as a public health concern that requires actions and regulations to control its prevalence worldwide (Khwaldeh et al., 2021). Unlike CS, in WPS, charcoal is used to burn the tobacco, and it has been reported that both charcoal and tobacco contain very potent toxicants such as heavy metals, tar, nicotine, carbon monoxide, polycyclic and heterocyclic aromatic hydrocarbons (Shihadeh et al., 2015).

Recently a cross-sectional study revealed the association of exclusive waterpipe smoking, with increased risk of coronary artery disease through the occurrence of calcification on arterial walls (Chami et al., 2019).

An augmentation in the heart rate, mean brachial arterial pressure, and acute arterial stiffness has been reported in waterpipe smokers compared with nonsmokers (Rezk-Hanna et al., 2018). Another clinical study revealed that chronic exposure to WPS was associated with a high risk of coronary artery disease (Sibai et al., 2014). Furthermore, a cross-sectional study demonstrated a strong association between WPS and heart disease (Islami et al., 2013).

Social smoking, also known as light smoking, or occasional smoking is a comprehensive term that indicates smoking on a nondaily basis (Wortley et al., 2003). This pattern of tobacco use is thought to have less or no adverse health effects compared with regular ones (Schane et al., 2010). A large epidemiological study showed that light cigarette smoking was associated with ischemic heart disease in both men and women (Bjartveit and Tverdal, 2005). A Surgeon General report has stated that three times higher the risk of dying from aortic aneurysm in light smokers compared with nonsmokers (Office of the Surgeon G et al., 2004). Moreover, it has been shown that occasional cigarette smoking increases cardiovascular mortality among men (Luoto et al., 2000). Reports have shown that, unlike cigarette smoking, the occasional use of WPS appears to be more prevalent in Eastern Mediterranean Region (Chaaya et al., 2004; Asfar et al., 2005).

We have recently shown that both occasional waterpipe smoking (Occ-WPS) and regular waterpipe smoking (Reg-WPS) were able to trigger prothrombotic events *in vivo* and *in vitro* and endothelial alterations (Hamadi et al., 2022). However, as far as we are aware, there is a paucity of experimental studies on the impact of long-term exposure to Occ-WPS versus Reg-WPS on cardiac injury, and the mechanisms underlying these effects. Therefore, the purpose of the present study is to evaluate in mice the effects and underlying mechanism of action of long-term exposure (6 months) to either Occ-WPS or Reg-WPS by assessing a comprehensive set of relevant cardiac endpoints including systolic blood pressure, cardiac injury, inflammation, oxidative stress, DNA damage, Nrf2 expression, and mitochondrial function.

## 2 Materials and methods

### 2.1 Animals and treatments

The project underwent review and approval by the Institutional Animal Care and Use Committee of the United Arab Emirates University (Approval # ERA_2017_5625), and the experiments were conducted following protocols approved by the Institutional Animal Care and Research Advisory Committee.

### 2.2 WPS exposure

Male BALB/C mice, aged 6–8 weeks and weighing 20–25 g, obtained from (animal house facility, College of Medicine and Health Sciences, United Arab Emirates University), were housed in the local central animal facility of the College of Medicine and Health Sciences. The mice were kept under controlled conditions, including a 12-h light-dark cycle, 60% humidity, and temperature of 22°C ± 1°C with free access to food and water.

After 1 week of acclimatization period to the experimental conditions, the mice were randomly divided into three groups: air (control), Occ-WPS, and Reg-WPS. The WPS exposure followed previously described methods (Nemmar et al., 2013; Nemmar et al., 2017). The mice were placed in soft restraints and connected to an exposure tower. Using a nose-only exposure system connected to a waterpipe device (inExpose System, SCIREQ, Canada), the mice were exposed to either air or WPS through their nasal passages. The WPS consisted of commercially available apple-flavored tobacco, generating mainstream WPS. Each daily session involved placing 10 g of tobacco into the WPS head, and any remaining tobacco was discarded at the end of the WPS exposure session.

The control mice were exposed to normal air only during the sessions, for 30 min/day, 5 days/week for 6 months. In the case of Occ-WPS group, mice were exposed to WPS, 30 min/day, 1 day/week for 6 months and for Reg-WPS group, mice were exposed to WPS, 30 min/day, 5 days/week for 6 months (Nemmar et al., 2013; Nemmar et al., 2017). The WPS exposure procedure was monitored by a computerized system that produces a computer-monitored puff every minute, consisting of a 2-s puff of WPS followed by 58 s of fresh air.

### 2.3 Systolic blood pressure (SBP) measurement

A non-invasive computerized tail-cuff manometry system (AD Instrument, Colorado Springs, CO, United States) was utilized to measure SBP (Ali et al., 2011; Nemmar et al., 2017). Before the actual experiment, the animals underwent a 3-day training period to minimize any anxiety caused by the measurement technique. We performed five measurements for each animal and took the average of the 5 readings. At the start of the experiment, and subsequently once a week throughout the study, SBP was measured.

### 2.4 Measurement of troponin I, brain natriuretic peptide (BNP), atrial natriuretic peptide (ANP), lactate dehydrogenase (LDH), creatine phosphokinase (CK), cotinine E-selectin, vascular cell adhesion molecule-1 (VCAM-1), intercellular adhesion molecule-1 (ICAM-1) and mammalian target of rapamycin (mTOR) concentrations in heart homogenates

The hearts that were collected from euthanized mice were rapidly rinsed with ice-cold PBS (pH 7.4) and then homogenized in a 0.1 M phosphate buffer (pH 7.4) containing 0.15 M KCl, 0.1 mM EDTA, 1 mM DTT, and 0.1 mM phenyl methyl sulfonyl fluoride at a temperature of 4°C. The homogenates were subjected to centrifugation at 14,000 rpm at 4°C for 20 min to remove cellular debris, and the resulting supernatants were frozen at −80°C until further biochemical analysis (Nemmar et al., 2017). The protein concentration in the homogenates was determined using the bicinchoninic acid assay. The activities of LDH and CK were measured spectrophotometrically using kits obtained from Roche (Basel, Switzerland). BNP, ANP and cotinine concentrations were assessed using an ELISA kit from My BioSource (San Diego, CA, United States) and Elabscience (Wuhan, Hubei, China) respectively. E-selectin, VCAM-1, and ICAM-1, mTOR concentrations were measured using ELISA Kits obtained from R & D Systems (Minneapolis, MN, United States).

### 2.5 Assessment of the levels of lipid peroxidation (LPO), reduced glutathione (GSH), catalase and superoxide dismutase (SOD) in heart homogenates

Measurement of GSH, catalase, and SOD levels was carried out using spectrophotometric method with commercially available kits (Cayman Chemical, Ann Arbor, Michigan, United States). NADPH-dependent membrane lipid peroxidation was quantified by measuring thiobarbituric acid reactive substances, using malondialdehyde as a standard (Sigma-Aldrich Fine Chemicals, St. Louis, MO, United States).

### 2.6 Measurement of interleukin-1β (IL-1β), tumor necrosis factor α (TNFα), interleukin-17 (IL17), monocyte chemoattractant protein-1 (MCP-1) and the chemokine CXCL1 concentrations in heart homogenates

Heart homogenates were prepared as described previously (Nemmar et al., 2017). The concentrations of IL-1β, TNF-α, IL17, MCP-1 and chemokine CXCL1 were determined by ELISA following the protocols provided by the vendor (R & D Systems, Minneapolis, MN, United States).

### 2.7 Evaluation of DNA damage in the heart by COMET assay

DNA damage was evaluated by using COMET assay, following the methodology described in our previous study (Nemmar et al., 2016a). Right after the animals sacrifice, the heart was extracted. Single-cell suspensions from the various hearts were acquired using the procedure outlined by de Souza et al (de Souza et al., 2014). Each harvested heart was rinsed in a cold medium consisting of RPMI 1640, 15% DMSO, and 1.8% (w/v) NaCl. The heart tissues were then placed in 1.5 mL of medium and finely chopped into pieces within a Petri dish using scissors. These pieces were allowed to settle, and the resulting liquid was collected in a 15 mL tube. The cell suspension obtained was subjected to centrifugation at 1000 rpm for 5 min at 4°C. The liquid above the pellet was removed, and the pellet was resuspended in 0.5 mL of the medium. Subsequently, the cell suspensions were mixed with a low melting point agarose solution (0.65%) and spread onto microscope slides pre-coated with 1.5% agarose. For each treatment, five slides were prepared. These slides were then incubated in ice-cold lysis buffer (composed of 2.5 M NaCl, 10 mM Tris, 100 mM EDTA, 1% Triton X-100, and 10% DMSO) at 4°C for at least 1 h to eliminate cell membranes. After this incubation, the slides were positioned in a horizontal electrophoresis unit and incubated in electrophoresis buffer (0.2 M EDTA, 5 M NaCl, pH 10) for 20 min to facilitate DNA unwinding and the expression of alkali-labile sites. Subsequently, electrophoresis was carried out for 20 min at 25 V and 300 mA. Following this, the slides were neutralized with Tris buffer (0.4 M Trizma base, pH 7.5) for 5 min and then washed with methanol. Finally, the slides were stained with propidium iodide, as previously described (Olive et al., 1994; Nemmar et al., 2016b). To avoid any further DNA damage, all of these procedures were conducted in the absence of light. The slides were affixed to a fluorescence microscope for cell scoring. Fifty cells from each treatment group were assessed, and their DNA migration was analyzed. The average of the five slides within each group was then computed. The measurement of the extent of DNA migration, specifically the diameter of the nucleus plus the migrated DNA, was determined using image analysis software Axiovision 3.1 (Carl Zeiss, Canada) (Hartmann and Speit, 1997; Owens and Peppas, 2006).

### 2.8 Quantification of p53 expression in heart tissue

Protein levels of p53 were assessed through Western blotting technique as described previously (Nemmar et al., 2018). Heart tissues obtained from the mice were immediately frozen in liquid nitrogen and stored at −80°C. Later, the tissues were weighed, rinsed with saline, and then homogenized with lysis buffer (pH 7.4). This buffer contained NaCl (140 mM), KCl (300 mM), trizma base (10 mM), EDTA (1 mM), Triton X-100 (0.5% v/v), sodium deoxycholate (0.5% w/v), and protease and phosphatase inhibitors. The homogenates were subjected to centrifugation for 20 min at 4°C. The resulting supernatants were collected, and protein concentration was determined using a Pierce bicinchoninic acid protein assay kit (Thermo Scientific, Waltham, MA, United States). A 35 µg sample of protein was electrophoretically separated by 10% sodium dodecyl sulfate polyacrylamide gel electrophoresis and then transferred onto polyvinylidene difluoride membranes. The immunoblots were blocked with 5% non-fat milk and then probed with polyclonal rabbit p53 antibody (dilution 1:1000, Abcam, Hong Kong, China) at 4°C overnight. Following this, the blots were incubated with a goat anti-rabbit IgG horseradish peroxidase-conjugated secondary antibody (dilution 1:5000, Abcam, Hong Kong, China) for 2 h at room temperature and visualized using the Pierce enhanced chemiluminescent plus Western blotting substrate Kit (Thermo Scientific, Waltham, MA, United States). Densitometric analysis of the protein bands for p53 was conducted using Typhoon FLA 9500 (GE Healthcare Bio-Sciences AB, Uppsala, Sweden). The blots were subsequently re-probed with a mouse monoclonal β-actin antibody (dilution 1:10,000, Santa Cruz Biotechnology, Dallas, Texas, United States) and used as a control.

### 2.9 Quantification of nuclear factor erythroid-derived 2-like 2 (Nrf2) expression in heart tissue

Five-micrometer heart sections were prepared and mounted on aminopropyltriethoxysilane (APES) coated slides. After dewaxing with xylene and rehydrating with graded alcohol, slides were placed in 0.01 M citrate buffer solution (pH = 6.0) and pre-treatment procedures to unmask the antigens was performed in a water bath at 95°C for 30 min. Then, sections were treated with peroxidase block for 60 min followed by protein block for 60 min. Sections were incubated with anti-Nrf2 (rabbit polyclonal, 1:100, Abcam, United States) for 1 hour at room temperature. After conjugation with primary antibodies, sections were incubated with secondary antibody (EnVisionTM Detection System, DAKO, Agilent, United States) for 20 min at room temperature followed by addition of DAB chromogen (EnVisionTM Detection System, DAKO, Agilent, United States) and counter staining done with hematoxylin. Appropriate positive controls were used. For negative control, the primary antibody was not added to sections. Positive and negative controls were used in every batch of slides that were stained (not shown in figures) (Nemmar et al., 2022).

### 2.10 Isolation of heart mitochondria and assessment of mitochondrial electron transport complexes

Mitochondria were isolated from the hearts of mice using differential centrifugation, following established protocols (Nemmar et al., 2023). In brief, the hearts were chopped and homogenized using a Dounce homogenizer in a mitochondrial isolation buffer composed of 0.32 M sucrose, 1 mM EDTA, and 10 mM Tris base. The resulting homogenate was subjected to centrifugation at 1000 rpm for 10 min at 4°C. The supernatants obtained from this step were collected and further centrifuged at 15,000 rpm for 15 min at 4°C. The resulting mitochondrial pellets were then resuspended in the mitochondrial isolation buffer and stored at temperatures below −80°C for future use. The activity of complex I, complex II & III and complex IV were determined using a previously established method (Nemmar et al., 2023).

### 2.11 Statistical analysis

Graphs were generated using GraphPad Prism Version 7 for Windows software (GraphPad Software Inc, San Diego, CA, United States). The data were presented as means ± SEM (standard error of the mean). The normality of the data was assessed by Shapiro-Wilk normality test. The data was analyzed by one-way analysis of variance (ANOVA), followed by Holm-Sidak’s multiple comparisons test. Thus, we have taken into consideration the comparisons air vs*.* Occ-WPS, air vs*.* Reg- WPS, and Occ-WPS vs*.* Reg-WPS for all the assessed parameters. Significance was defined as *p* ≤ 0.05.

## 3 Results

### 3.1 Effect of Occ-WPS or Reg-WPS exposure on SBP

**FIGURE 1:**
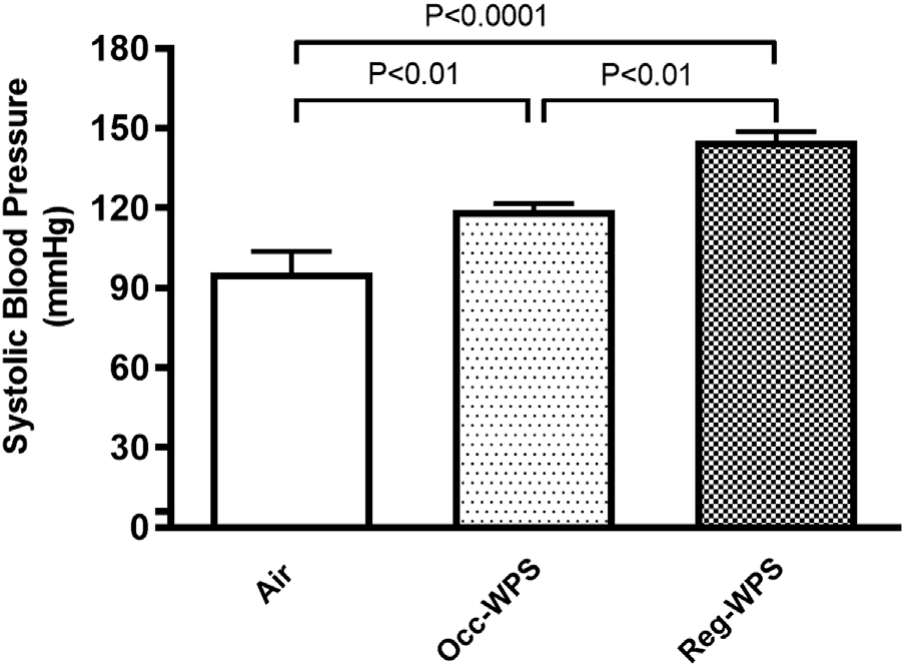
Systolic blood pressure (SBP) at the end of the 6 months exposure period to air (control) or occasional waterpipe smoke (Occ-WPS) or regular WPS (Reg-WPS). Data are mean ± SEM (*n* = 7–8).

Figure 1 illustrates the impact of 6 months of exposure to either Occ-WPS or Reg-WPS on SBP in mice. The exposure to either Occ-WPS or Reg-WPS induced a statistically significant increase in SBP (*p* < 0.01) and (*p* < 0.0001) respectively compared with air-exposed mice. Furthermore, there was a statistical significance (*p* < 0.01) between Occ-WPS and Reg-WPS groups.

### 3.2 Effect of Occ-WPS or Reg-WPS exposure on troponin I, BNP, ANP, LDH, and CK levels in heart homogenates

**FIGURE 2:**
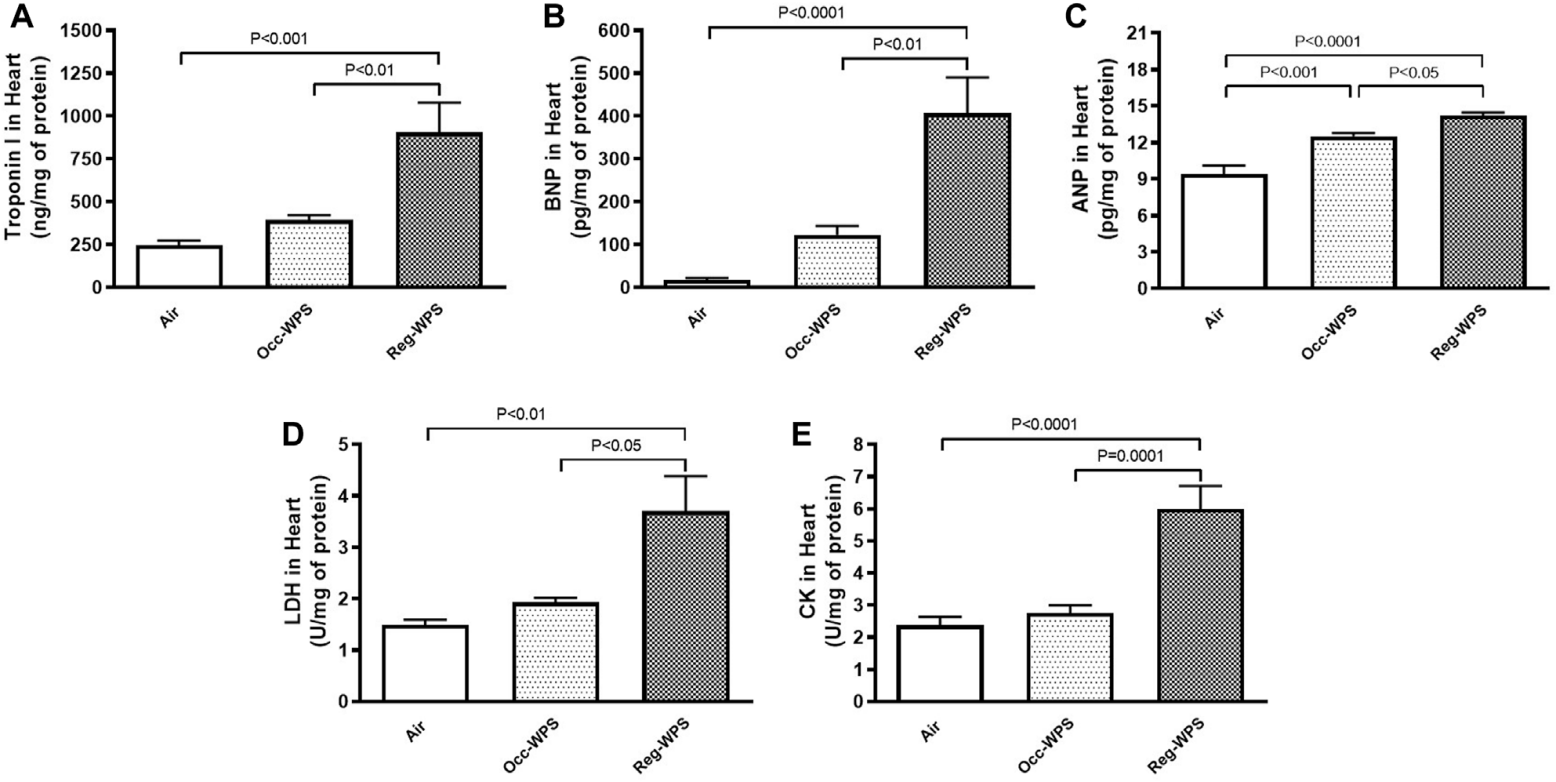
Troponin I **(A)**, brain natriuretic peptide BNP, **(B)**, atrial natriuretic peptide ANP, **(C)**, lactate dehydrogenase LDH, **(D)**, and creatine phosphokinase CK, **(E)** levels in the heart of mice at the end of the 6 months exposure period to air (control) or occasional waterpipe smoke (Occ-WPS) or regular WPS (Reg-WPS). Data are mean ± SEM (*n* = 7–8).

The effect of either Occ-WPS or Reg-WPS on the cardiac concentrations of troponin I (Figure 2A), BNP (Figure 2B), ANP (Figure 2C), LDH (Figure 2D), and CK (Figure 2E) was evaluated by ELISA. Our results showed no significant augmentation in troponin I, BNP, LDH, and CK levels in heart homogenates of mice occasionally exposed to WPS compared with air-exposed group except for ANP where we recorded a significant increase (*p* < 0.001). In contrast, regular exposure to WPS caused a significant elevation in the levels of troponin I (*p* < 0.001), BNP (*p* < 0.0001), ANP (*p* < 0.0001), LDH (*p* < 0.01), and CK (*p* < 0.0001) compared with air-exposed group. Moreover, there was a statistical significance between Occ-WPS and Reg-WPS groups in the levels of troponin I (*p* < 0.01), BNP (*p* < 0.01), ANP (*p* < 0.05), LDH (*p* < 0.05), and CK (*p* = 0.0001).

### 3.3 Effect of Occ-WPS or Reg-WPS exposure on LPO, GSH, catalase and SOD levels in heart homogenates

**FIGURE 3:**
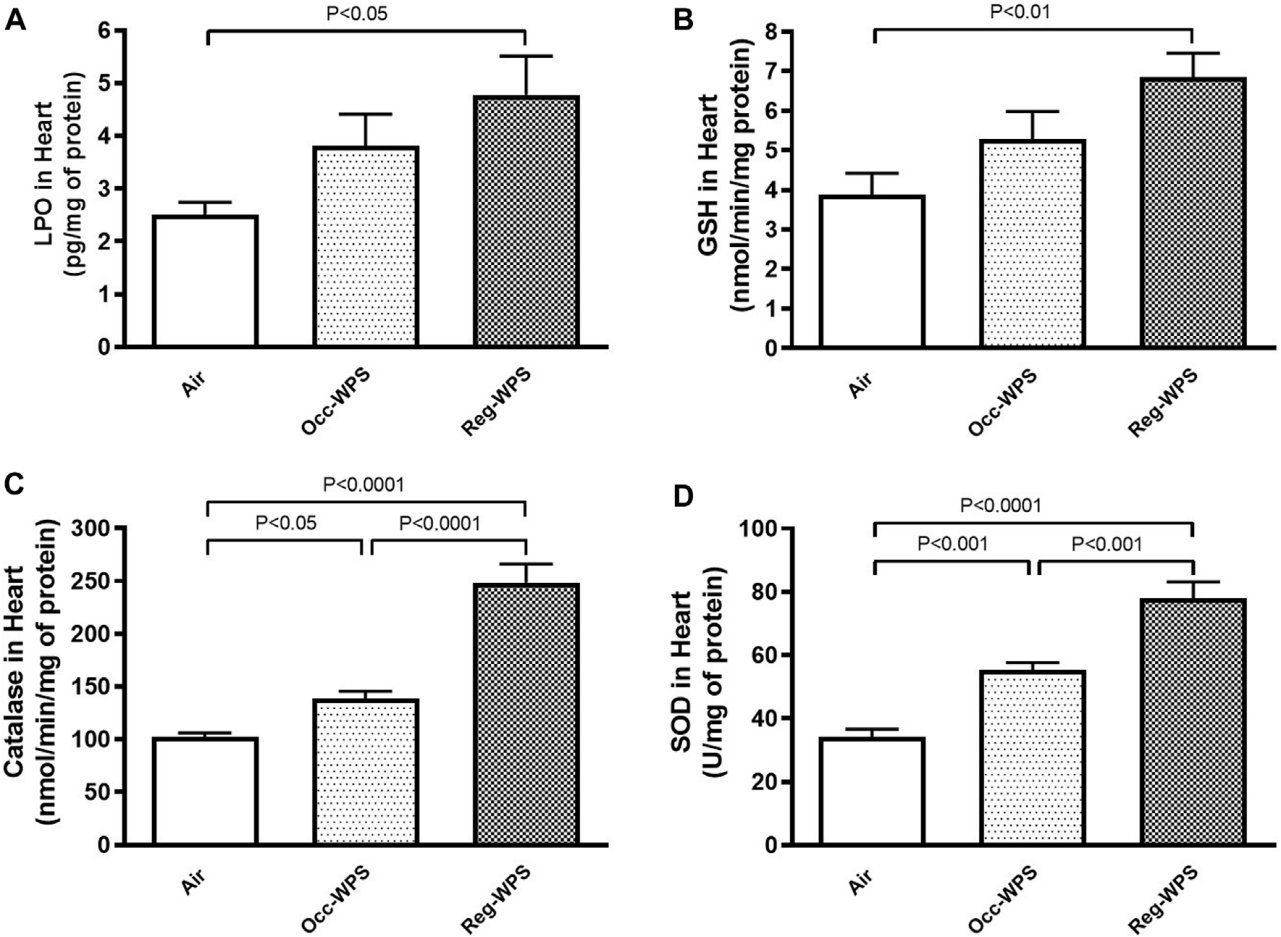
Lipid peroxidation LPO, **(A)**, reduced glutathione GSH, **(B)**, catalase **(C)** and superoxide dismutase SOD, **(D)** levels in the heart of mice at the end of the 6 months exposure period to air (control) or occasional waterpipe smoke (Occ-WPS) or regular WPS (Reg-WPS). Data are mean ± SEM (*n* = 7–8).

The measurement of the levels of oxidative markers following either Occ-WPS or Reg-WPS exposure is illustrated in Figure 3. The concentrations of the LPO and GSH concentrations in heart homogenates were slightly increased following Occ-WPS compared with air-exposed group but this increase did not reach a statistical significance. Moreover, the exposure to Occ-WPS caused a significant increase in catalase (*p* < 0.05) and SOD (*p* < 0.001). Reg-WPS induced a significant increase in the levels of LPO (*p* < 0.05), GSH (*p* < 0.01), catalase (*p* < 0.0001) and SOD (*p* < 0.0001) compared with air-exposed group. In addition, there was statistical significance in catalase (*p* < 0.0001) and SOD (*p* < 0.001) between Occ-WPS and Reg-WPS groups but not in LPO and GSH.

### 3.4 Effect of Occ-WPS or Reg-WPS exposure on MCP-1 and the chemokine CXCL1 concentrations in heart homogenates

**FIGURE 4:**
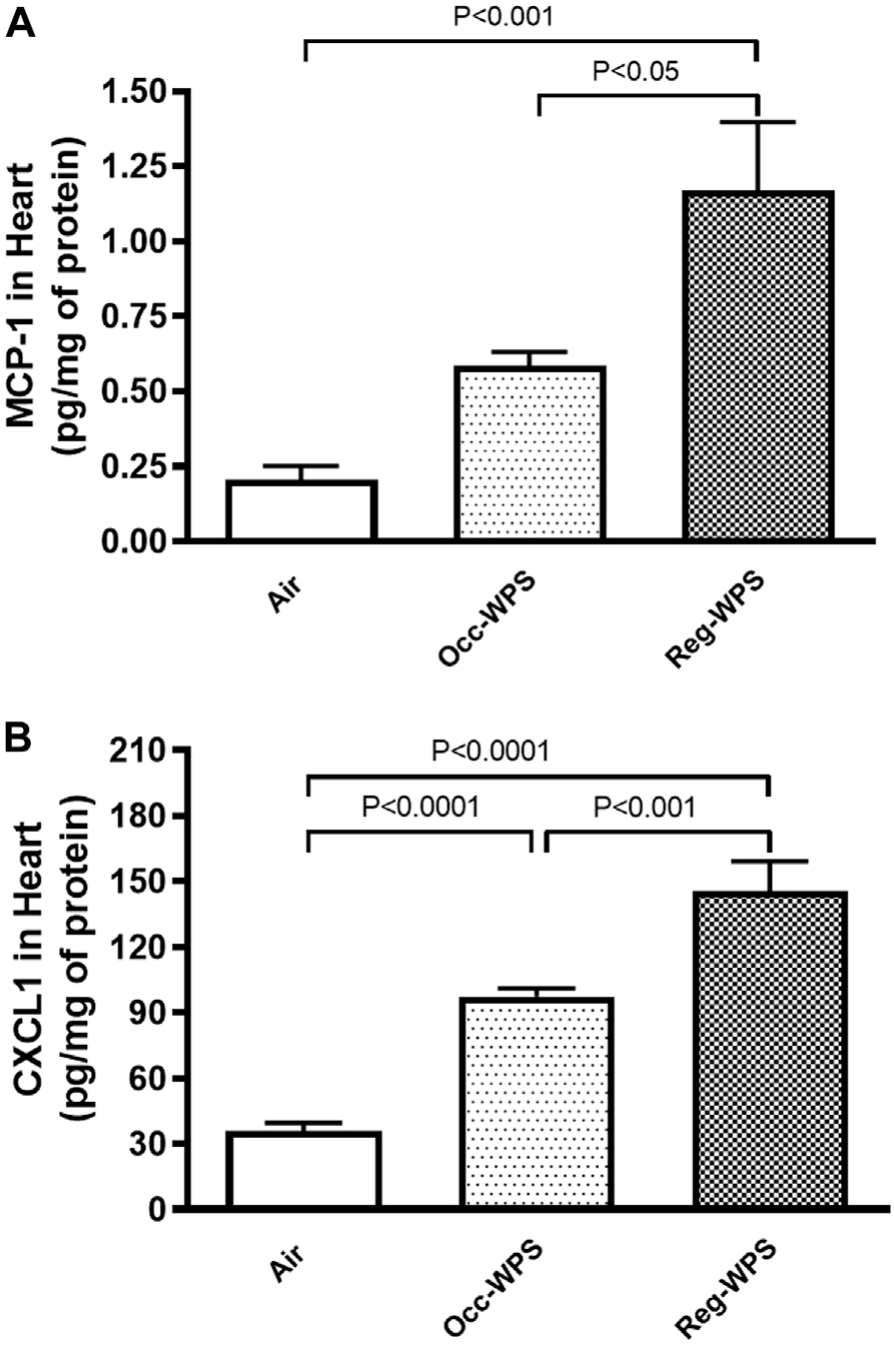
Monocyte chemoattractant protein-1 MCP-1, **(A)** and chemokine CXCL1 **(B)** concentrations in the heart of mice at the end of the 6 months exposure period to air (control) or occasional waterpipe smoke (Occ-WPS) or regular WPS (Reg-WPS). Data are mean ± SEM (*n* = 7–8).

Figure 4 illustrates the effect of exposure to either Occ-WPS or Reg-WPS for 6 months on the concentrations of MCP-1 (A) and the chemokine CXCL1 (B) compared with air-exposed group. Exposure to Occ-WPS augmented significantly the concentrations of CXCL1 (*p* < 0.0001) but did not reach statistical significance for MCP-1. However, Reg-WPS induced a significant increase in the concentrations of both MCP-1 (*p* < 0.001) and CXCL1 (*p* < 0.0001). Furthermore, there was statistical significance between Occ-WPS and Reg-WPS groups in the concentration of both MCP-1 (*p* < 0.05) and CXCL1 (*p* < 0.001).

### 3.5 Effect of Occ-WPS or Reg-WPS exposure on E-selectin, VCAM-1 and ICAM-1 concentrations in heart homogenates

**FIGURE 5:**
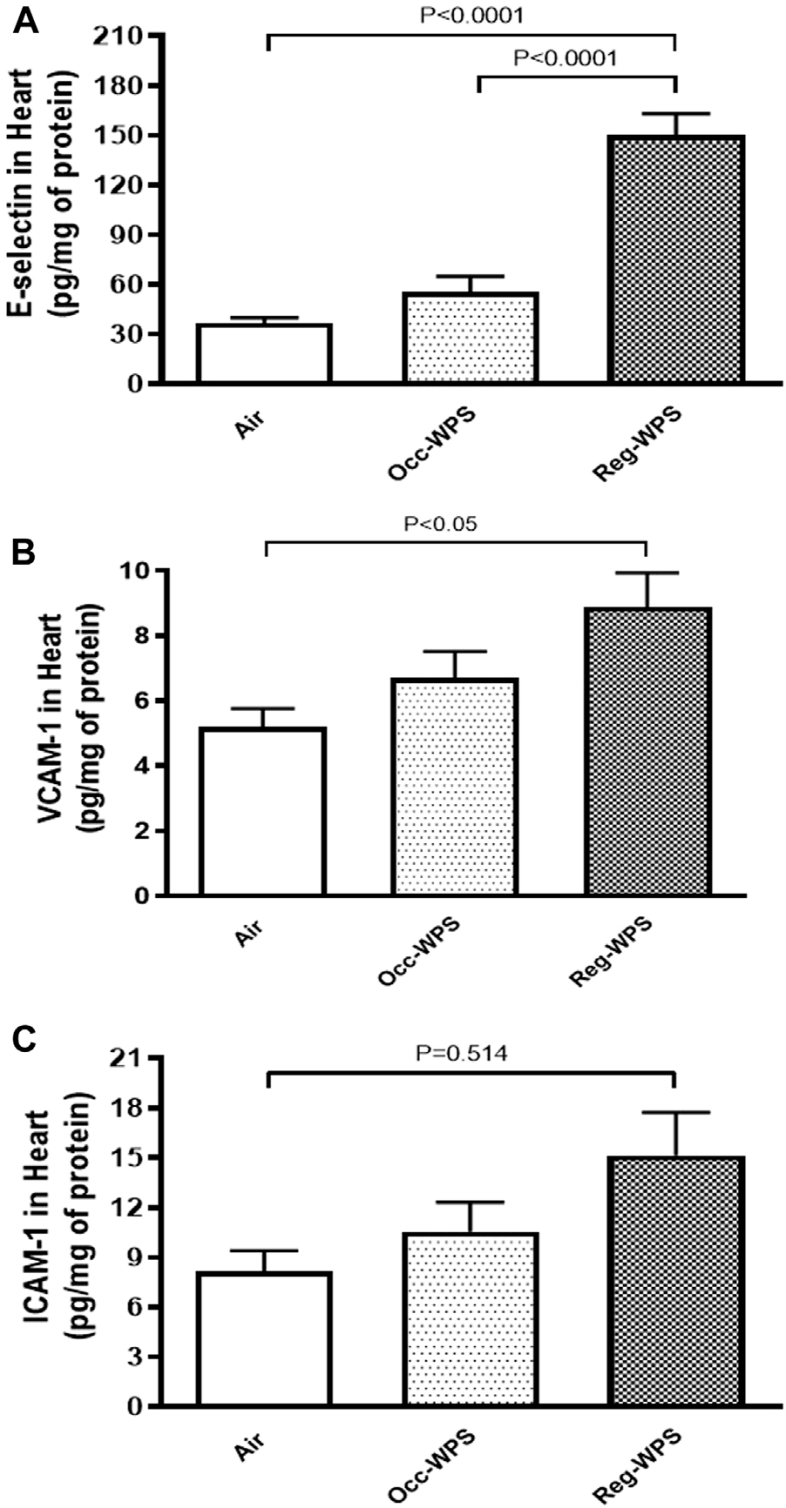
E-selectin **(A)**, vascular cell adhesion molecule-1 VCAM-1, **(B)**, and intercellular adhesion molecule-1 ICAM-1, **(C)** concentrations in the heart of mice at the end of the 6 months exposure period to air (control) or occasional waterpipe smoke (Occ-WPS) or regular WPS (Reg-WPS). Data are mean ± SEM (*n* = 7–8).

The E-selectin, VCAM-1 and ICAM-1 concentrations in heart homogenates were not significantly augmented after Occ-WPS exposure for a period of 6 months compared with air-exposed group. However, we observed a significant increase in the concentrations of E-selectin (*p* < 0.0001, Figure 5A), VCAM-1 (*p* < 0.05, Figure 5B), and ICAM-1 (*p* = 0.514, Figure 5C) following exposure to Reg-WPS. Moreover, there was statistical significance between Occ-WPS and Reg-WPS groups only in the concentration of E-selectin (*p* < 0.0001).

### 3.6 Effect of Occ-WPS or Reg-WPS exposure on IL1β, TNFα and IL17 concentrations in heart homogenates

**FIGURE 6:**
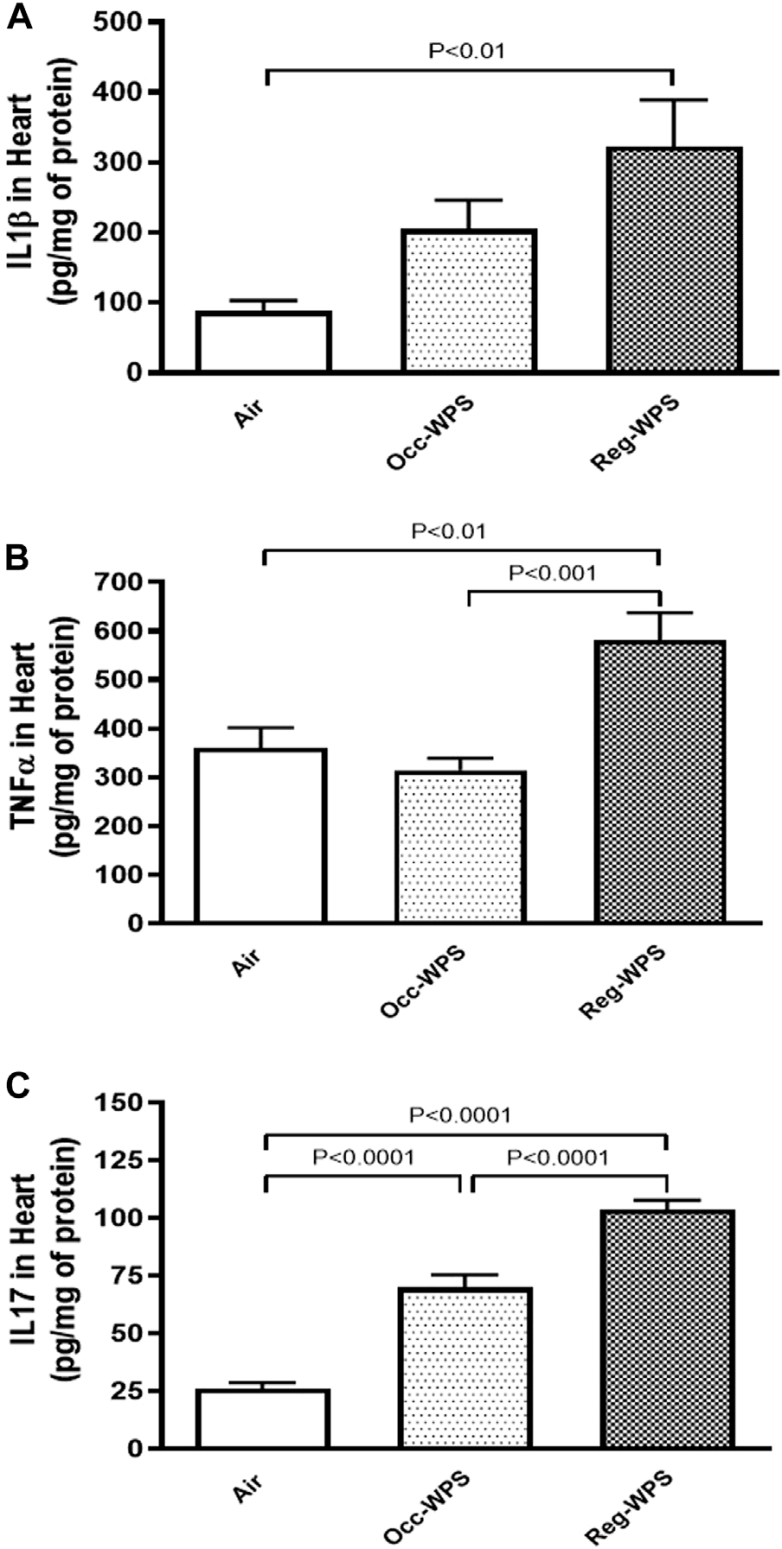
Interleukin-1β IL-1β, **(A)**, tumor necrosis factor α TNFα, **(B)** and interleukin 17 IL17, **(C)** concentrations in the heart of mice at the end of the 6 months exposure period to air (control) or occasional waterpipe smoke (Occ-WPS) or regular WPS (Reg-WPS). Data are mean ± SEM (*n* = 7–8).

Figure 6 shows the effect of exposure to either Occ-WPS or Reg-WPS for 6 months on IL-1β (A), TNFα (B) and IL17 (C), compared with air-exposed group. Occ-WPS caused a significant change only in the concentrations of IL17 (*p* < 0.0001) but not for IL1β and TNFα. However, Reg-WPS induced a significant increase in in the concentrations of IL-1β (*p* < 0.01), TNFα (*p* < 0.01) and IL17 (*p* < 0.0001). Furthermore, there was statistical significance between Occ-WPS and Reg-WPS groups in the concentrations of TNFα (*p* < 0.001) and IL17 (*p* < 0.0001) but not in IL-1β.

### 3.7 Effect of Occ-WPS or Reg-WPS exposure on DNA migration in heart homogenate

**FIGURE 7:**
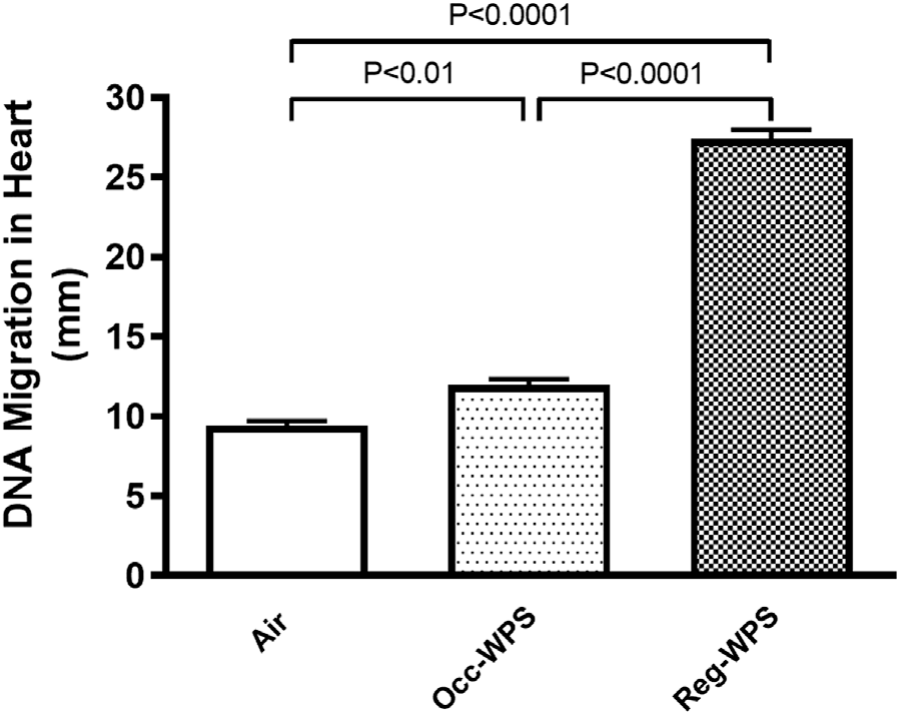
DNA migration was assessed by COMET assay in heart tissues of mice at the end of the 6 months exposure period to air (control) or occasional waterpipe smoke (Occ-WPS) or regular WPS (Reg-WPS). Data are mean ± SEM (*n* = 7–8).

Figure 7 shows that both exposure to Occ-WPS and Reg-WPS for 6 months induced cardiac DNA injury (*p* < 0.01) and (*p* < 0.0001) respectively compared with air-exposed group. Furthermore, there was statistical significance (*p* < 0.0001) between Occ-WPS and Reg-WPS groups.

### 3.8 Effect of Occ-WPS or Reg-WPS exposure on mTOR and p53 concentrations in heart homogenates

**FIGURE 8:**
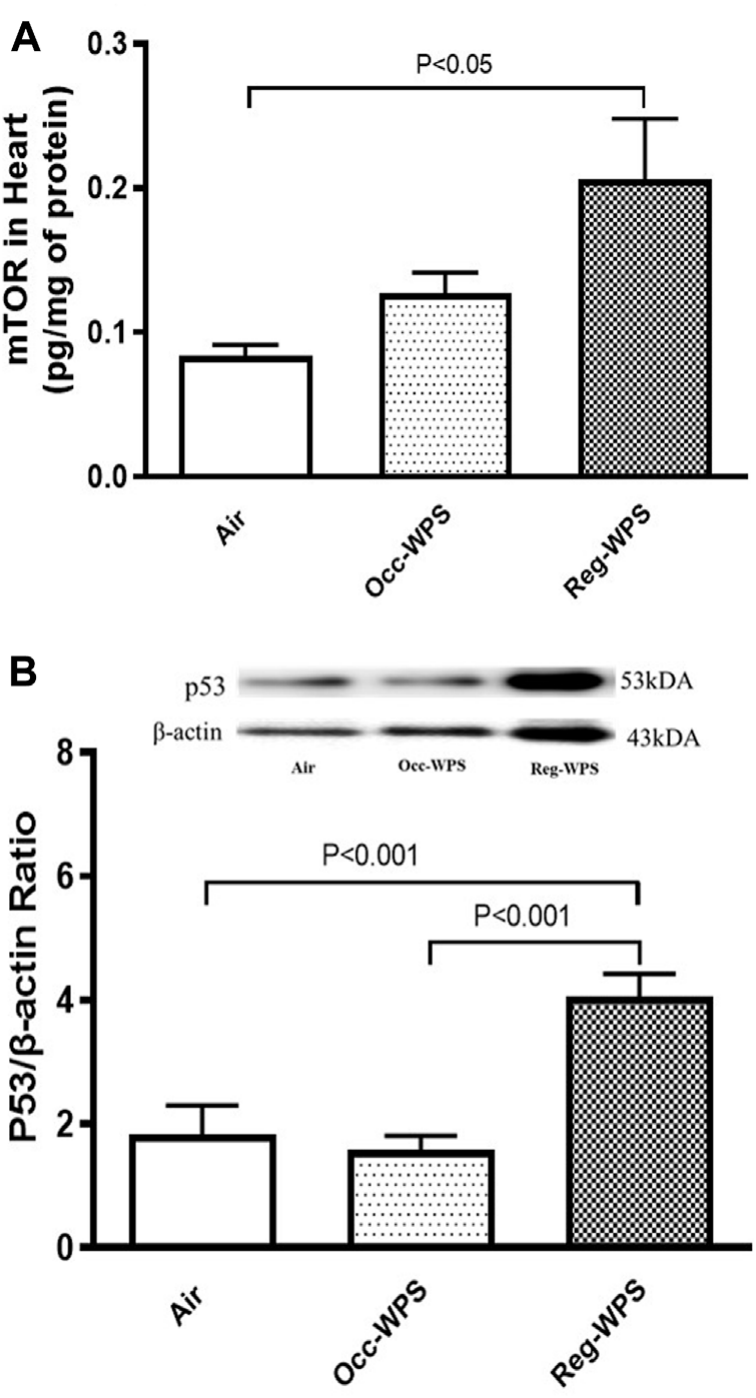
Mammalian target of rapamycin (mTOR) **(A)** and p53 **(B)** levels in heart tissues of mice at the end of the 6 months exposure period to air (control) or occasional waterpipe smoke (Occ-WPS) or regular WPS (Reg-WPS). Data are mean ± SEM (*n* = 7–8).

Figure 8 exemplifies the heart homogenate levels of mTOR and p53. While the inhalation of Occ-WPS for 6 months did not increase significantly the concentration of mTOR and p53, Reg-WPS induced a significant elevation in the levels of both mTOR (*p* < 0.05) and p53 (*p* < 0.001) compared with air-exposed group. In addition, there was a statistical significance in the levels of p53 (*p* < 0.001) between Occ-WPS and Reg-WPS groups.

### 3.9 Effect of Occ-WPS or Reg-WPS exposure on Nrf2 expression in heart tissue

**FIGURE 9:**
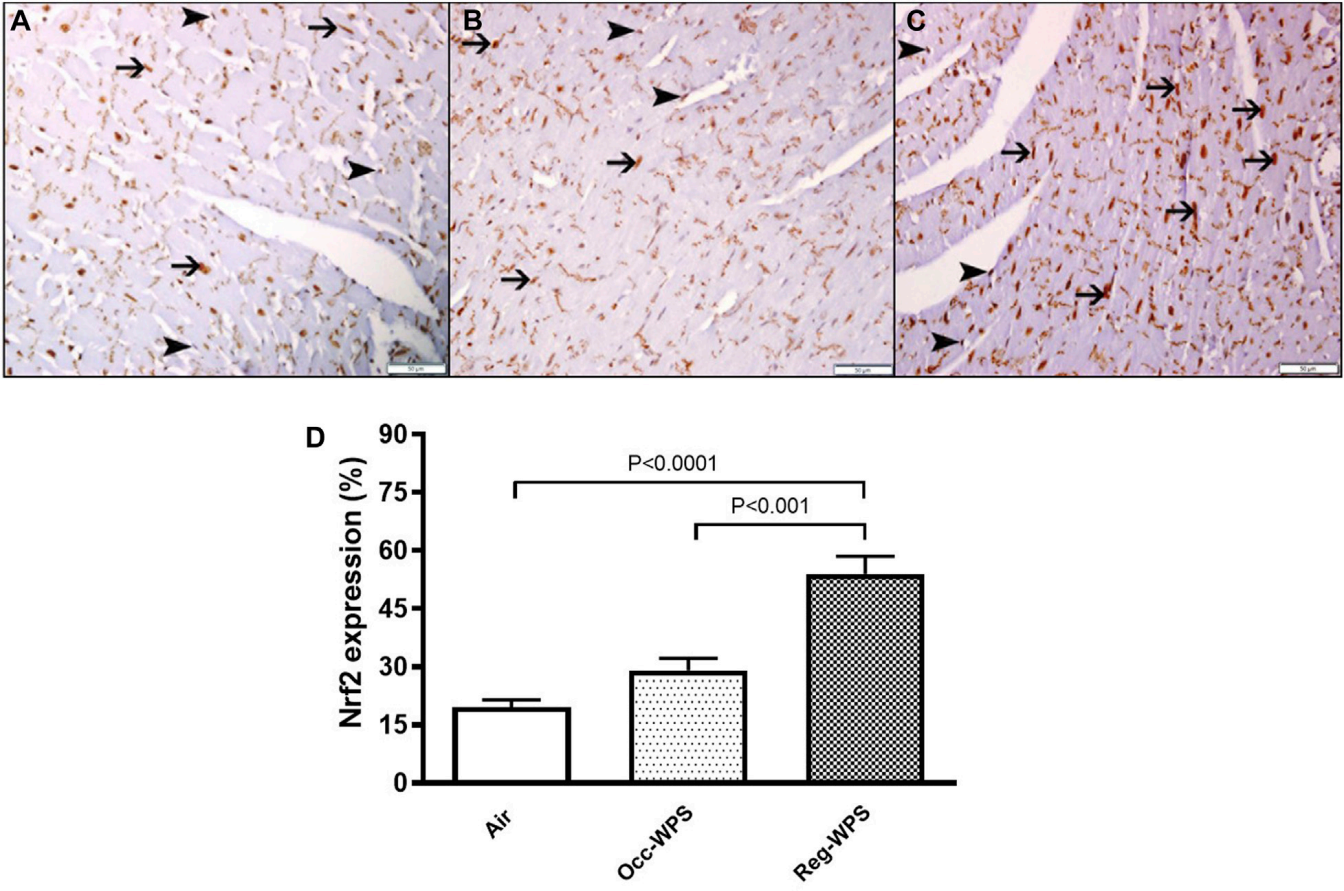
Immunohistochemical staining of the heart tissue sections of mice for the detection of nuclear factor erythroid-derived 2-like 2 (Nrf2) at the end of the 6 months exposure period to air (control) or occasional waterpipe smoke (Occ-WPS) or regular WPS (Reg-WPS). **(A)** representative section of the heart of air-exposed group showing focal mild expression of Nrf2 by cardiac myocytes (thin arrow) and endothelial cells (arrow head). **(B)** representative section of the heart of Occ-WPS exposure showing expression of Nrf2 by endothelial cells (arrow head) and cardiac myocytes (thin arrow). **(C)** representative section of the heart of Reg-WPS exposure showing expression of Nrf2 by endothelial cells (arrow head) and cardiac myocytes (thin arrow). **(D)** Semiquantitative quantification of the % of immunohistochemical assessment of the heart tissue for Nrf2 of air, Occ-WPS or Reg-WPS groups. Data are mean ± SEM (*n* = 6). Scale bars: 50 μm.

There was a expression of Nrf2 by cardiac myocytes and endothelial cells in the heart sections of all groups (Figures 9A–C), with different expression intensities and distributions. The 6 months air-exposed group shows a mild expression of Nrf2 by cardiomyocytes and endothelial cells and scored 1 (Figures 9A, D). The Occ-WPS group shows a non-significant augmentation in the expression of Nrf2 by cardiomyocyte and endothelial cells when compared with air mice scored 2 (Figures 9B, D). The Reg-WPS exhibits a significant increase (*p* < 0.0001) in the expression of Nrf2 by cardiomyocytes and endothelial cells when compared to air-exposed group and scored 3 (Figures 9C, D). Moreover, there was statistical significance (*p* < 0.001) between Occ-WPS and Reg-WPS groups.

### 3.10 Effect of Occ-WPS or Reg-WPS exposure on mitochondrial complexes I, II & III, IV activities in heart homogenates

**FIGURE 10:**
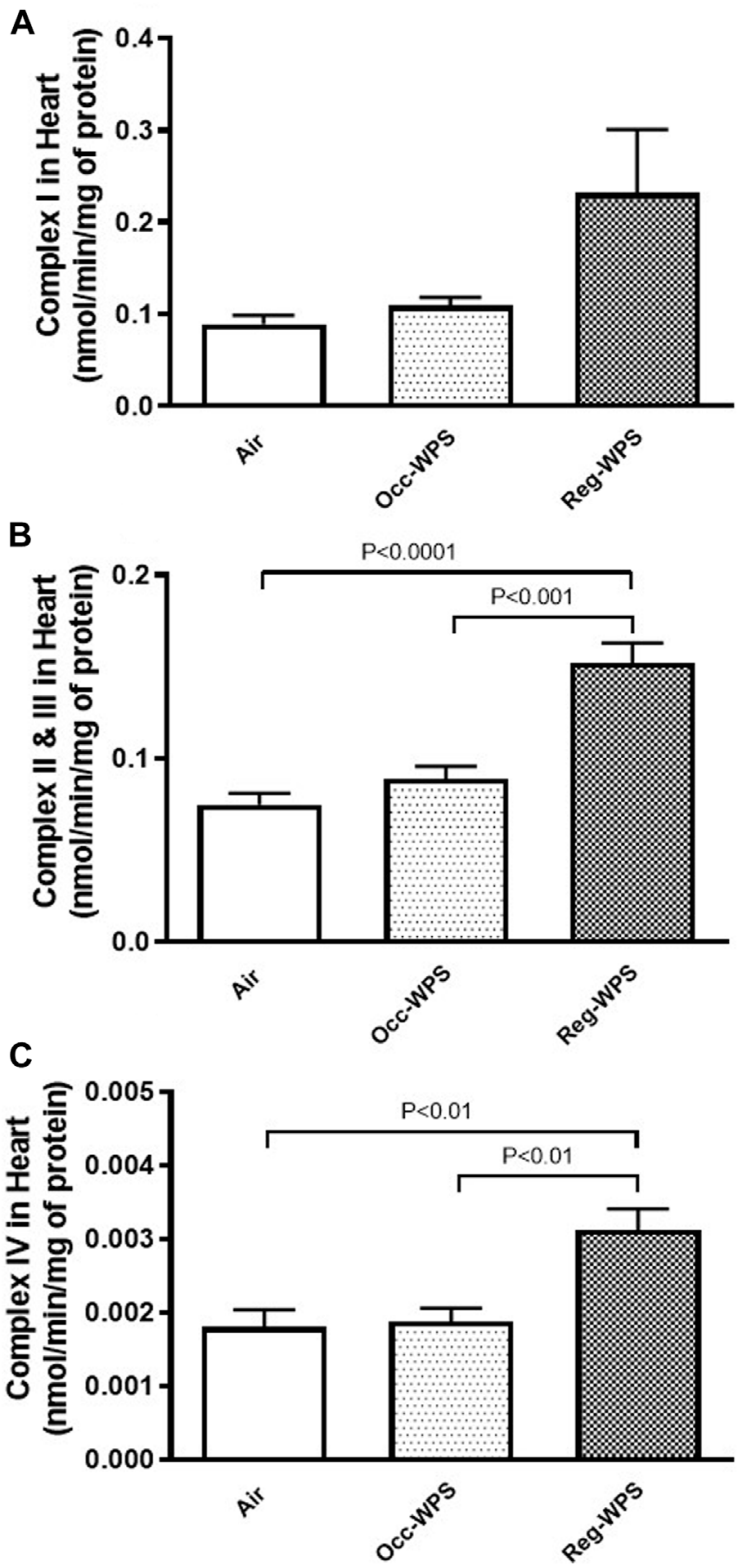
Mitochondrial complexes I **(A)**, II& III **(B)** and IV **(C)** activities in heart tissues of mice at the end of the 6 months exposure period to air (control) or occasional waterpipe smoke (Occ-WPS) or regular WPS (Reg-WPS). Data are mean ± SEM (*n* = 7–8).

As illustrated in Figures 10A–C, the cardiac activities of complexes I (A), II & III (B), and IV (C) were not impacted significantly in Occ-WPS mice exposed to WPS compared with air group. However, there was a significant augmentation in the activities of complexes II & III (*p* < 0.0001) and IV (*p* < 0.01) in Reg-WPS mice exposed to air compared with air-exposed mice. Additionally, there was statistical significance between Occ-WPS and Reg-WPS groups in the activities of complexes II & III (*p* < 0.001) and IV (*p* < 0.01).

### 3.11 Effect of Occ-WPS or Reg-WPS exposure on cotinine concentrations in plasma

**FIGURE 11:**
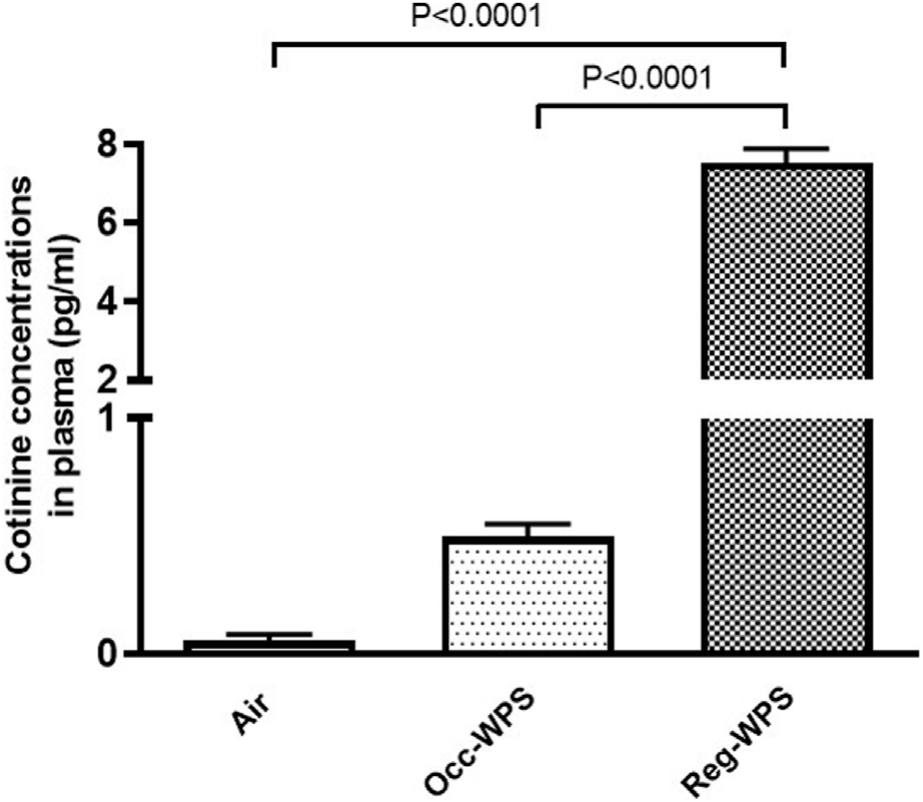
Cotinine concentrations measured in the plasma of mice at the end of the 6 months exposure period to air (control) or occasional waterpipe smoke (Occ-WPS) or regular WPS (Reg-WPS). Data are mean ± SEM (*n* = 7–8).

Figure 11 shows that exposure to Reg-WPS for 6 months induced a significant increase in the cotinine concentrations in plasma (*p* < 0.0001) compared with air-exposed group. Also, the concentration of cotinine in the plasma of Occ-WPS group increased but this increase did not reach statistical significance (*p* = 0.153). Furthermore, there was a statistical significance (*p* < 0.0001) between Occ-WPS and Reg-WPS groups.

## 4 Discussion

The aim of the present study is to evaluate the effect of chronic exposure to either Occ-WPS or Reg-WPS on the heart of mice and the related changes in the pathological parameters. We showed that Reg-WPS causes increase of SBP and cardiotoxicity evidenced by the elevation of markers of heart injury, inflammation, and oxidative markers. In addition, it induced cardiac DNA damage and mitochondrial dysfunction. Moreover, we found that Occ-WPS had a significant effect on SBP, ANP, antioxidant enzymes, IL17, the chemokine CXCL1 and cardiac DNA injury.

Our data show that either Occ-WPS or Reg-WPS exposure for 6mo caused an increase in the SBP compared with air-exposed mice, this effect was more pronounced and statistically significant in mice-exposed to Reg-WPS. Consistent with this observation, we and others, have reported that exposure to WPS mice or rats increases SBP (Nemmar et al., 2013; Nemmar et al., 2017; Nemmar et al., 2019; Nemmar et al., 2023). These findings strongly suggest that there are no safe levels of WPS on blood pressure. Additionally, we have recently shown that both Occ-WPS and Reg-WPS activate blood platelets, which increases the risk of thrombosis and damages the cellular lining of the arteries, and subsequently promotes atherosclerosis (Hamadi et al., 2022). In line with the notion that occasional WPS is considered as low-intensity smoking, a clinical study has reported that low cigarette consumption increases the risk of coronary heart disease. This might be attributed to the presence of nicotine and fine particulate matter in tobacco smoke, as well as the increased sympathetic nerve activity (Middlekauff et al., 2014). Furthermore, nicotine’s dual impact on inflammatory pathways through various nicotinic acetylcholine receptors can alter the inflammatory pathways, potentially resulting in excessive inflammation and the onset of hypertension (Wang et al., 2003; Wang et al., 2012).

Troponins are a group of proteins in the heart that regulate muscular contraction through controlling the calcium-mediated interaction between actin and myosin. Troponins are considered as subclinical myocardial injury markers that are linked to the risk of cardiovascular diseases (Jishi et al., 2004; Sharma et al., 2004). Our results showed that Occ-WPS induced a non-significant effect on the concentration of troponin I. However, Reg-WPS induced a significant elevation in the concentration of troponin I in the heart homogenates. This finding is in agreement with the recent report which showed that WPS induced an increase in serum cardiac troponin I in mice following exposure to WPS (Alavi et al., 2021). In the present study, we found that only chronic exposure to Reg-WPS led to a significant increase in the concentrations of BNP. These results are consistent with the clinical examination that revealed the levels of serum NT-pro-BNP were significantly higher in current CS smokers than in never smokers (Otsuka et al., 2010). Moreover, it has been shown that WPS deregulated several cardiac markers in Zebra fish including ANP and BNP (Zakaria et al., 2021). These results clearly indicate that there is an increased cardiac overload in Reg-WPS which is consistent with the fact that smoking in general promotes the risk of cardiovascular disease (Benowitz, 2003; Gallucci et al., 2020). Furthermore, a large clinical study consisting of almost 10,000 participants showed greater levels of NT-proBNP and troponin in active smokers compared with non-smokers (Nadruz et al., 2016). The main role of ANP is to stimulate the excretion of sodium and water in the kidneys and induce the relaxation of vascular smooth muscles. This action helps in controlling blood volume and blood pressure (Zou et al., 2020). Moreover, in the heart, independent to its effect on blood pressure it functions through both paracrine and autocrine mechanisms where it inhibits hypertrophy, fibrosis, arrhythmias, and cardiomyopathies, thus actively working to prevent the onset and advancement of heart failure (Al-Arifi et al., 2012). Our data show that either Occ-WPS or Reg-WPS exposure for 6mo induced an elevation in the concentration of ANP which indicates an occurrence of heart injury. In line with this, sub-chronic exposure to CS showed cardiac ANP and BNP genes were significantly elevated 14 and 4 fold respectively (Al-Arifi et al., 2012). Similar to the effect of WPS on troponin I and BNP, chronic exposure to Reg-WPS induced a significant augmentation in LDH activity and these results corroborate with the clinical findings that showed higher LDH levels in the saliva of e-cigarette users compared with controls (Pandarathodiyil et al., 2021). Cell death and tissue damage are pathological events that lead to the secretion of LDH extracellularly which increases its levels subsequently as an indication of cardiac damage and acute myocardial infarction (Macdonald et al., 1957). In the present study, we measured the concentrations of CK, an enzyme that promotes vascular contractility and has been lately considered as a new risk factor for hypertension (Brewster et al., 2019). The current data show that Reg-WPS triggered a significant increase in the levels of CK.

Oxidative stress takes place within the cell as a result of an imbalance between reactive oxygen species generation and antioxidant defense mechanisms (Zou et al., 2020). It is well known that in response to WPS, an excessive production of ROS occurs which can cause lipid peroxidation and DNA damage (Holmström and Finkel, 2014). In the current study, we have examined the cardiac levels of LPO, a marker of lipid peroxidation used as an index for oxidative stress. Likewise, the same effect was recorded with the GSH concentrations in the heart homogenates. Our results revealed that while Reg-WPS caused a significant increase in the levels of LPO, GSH, catalase and SOD, Occ-WPS triggered a statistically significant increase in the activities of both antioxidant enzymes catalase and SOD. These results are an indication of an oxidative stress state and are in agreement with previous experimental study that showed the occurrence of oxidative stress following WPS exposure (Nakhaee et al., 2019). Moreover, recently we have reported that exposure to WPS induced cardiac oxidative stress characterized by an increase in the activities of both catalase and SOD in the heart and plasma respectively (Nemmar et al., 2019; Nemmar et al., 2023).

The recruitment of immune cells from the blood to the tissue is mediated by contact through adhesion molecules. This interaction is mainly influenced by chemokines such as MCP-1 and CXCL1 (Papadopoulou et al., 2008; Korbecki et al., 2022). Our data show that Occ-WPS induced a significant elevation in the concentrations of CXCL1. However, Reg-WPS caused a significant augmentation in the concentrations of both MCP-1 and CXCL1. Exposure to passive smoke has been reported to raise MCP-1 levels in mice (Zhu et al., 2003). The incubation of blood from smokers without any external stimulation, elevates the concentrations of several chemokines, including IL-8 (Elisia et al., 2020). Additionally, *in vitro* study has shown that nicotine stimulates the production of MCP-1 by neutrophils and fibroblasts (Almasri et al., 2007).

There is a consistent association between CS consumption and elevated sICAM levels (Csordas et al., 2008). In the current study, our data indicated that the concentrations of the adhesion molecules E-selectin, VCAM-1, and ICAM-1 in the heart homogenates were significantly elevated following exposure to Reg-WPS. Our results corroborate a previous study that found that the concentrations of these adhesion molecules are increased by CS (Cavusoglu et al., 2004). It has been suggested that alterations in the levels of these adhesion molecules lead to stress on the vasculature and subsequently caused a diminution in the coronary blood flow as well as myocardial oxygen delivery (Deanfield and Selwyn, 1986; Brunner et al., 2005). In addition, *in vitro* study has shown that human umbilical vein endothelial cells exposed to CS extract induced the release of P-selectin/CD62P, IL-6, and IL-8 from endothelial cells into the supernatant (Bernhard et al., 2005). Remarkably, Blann and colleagues (Blann et al., 1997) demonstrated that regular smokers exhibit greater levels of sICAM and P-selectin, and these levels were decreased after a 6-week of smoking cessation which suggests the direct effect of cigarette smoking on such adhesion molecules.

To gain further insight into the pathophysiological mechanisms related to WPS-induced cardiotoxicity, we investigated the effect of chronic exposure to Occ-WPS or Reg-WPS on the proinflammatory cytokines. Inflammation in the heart is one of the hallmarks of exposure to CS. Our data show IL1β, TNFα and IL17 concentrations were significantly augmented in the Reg-WPS but only IL17 was significantly augmented in Occ-WPS in heart homogenates. These results corroborate previous findings that showed that exposure to regular WPS elevated IL1β, IL6, and TNF-α levels in the heart (Nakhaee et al., 2019; Nemmar et al., 2019; Nemmar et al., 2023). Moreover, *in vivo* study has shown that exposure to WPS condensate increased the gene expression of IL1β and IL6 in lung cells (Zaarour et al., 2021).

Overall, the results obtained in the present study show a more substantial increase in the cardiac markers of cytotoxicity, injury, oxidative stress, adhesion molecules, and inflammation. The latter might be attributed to the longer duration and hence the intensity of repeated exposure to WPS as compared with Occ-WPS which implies a more regular inhalation of toxicants present in WPS such as nicotine, tar, carbon monoxide, polycyclic aromatic hydrocarbons, nitrosamines, volatile aldehydes, phenols and heavy metals, catechol and hydroquinone (Schubert et al., 2015; Shihadeh et al., 2015).The current study also revealed that exposure to either Occ-WPS or Reg-WPS significantly increased cardiac DNA damage which shows that even Occ-WPS has a deleterious effect on the genetic material of the heart. A clinical study has confirmed the genotoxic effect of both WPS and CS (Alsaad et al., 2019). In line with latter study, the examination of lymphocytes collected from WPS smokers showed an aberrant DNA compared with nonsmokers (Alsatari et al., 2012).

The role of mTOR in innate immunity has been elucidated, highlighting its capacity to restrict proinflammatory agents (Weichhart et al., 2011). Hence, the mTOR has emerged as a critical controller of innate immunity (Säemann et al., 2009). Inhibiting mTOR with rapamycin enhances the production of TNF-α and IL-6 (Weichhart et al., 2008). Moreover, cardiac mTOR has been reported to act as a major regulator of oxidative stress through the promotion of mitochondrial biogenesis and oxidative metabolism (Zhao et al., 2017). Furthermore, mTOR has been reported to exert a protective effect against oxidative injury-induced endothelial dysfunction and cardiomyocyte toxicity (Wagner et al., 2010; Zhao et al., 2017). In the current study, our results revealed that Reg-WPS exposure caused an elevation in mTOR concentrations. However, a non-significant increase was found in the Occ-WPS group. In line with our results, an *in vivo* study showed mTOR was upregulated by tobacco extracts in the gefitinib-resistant cell lines PC9/GR and HCC827/GR (Zou et al., 2020). Furthermore, our results showed that chronic exposure to Reg-WPS induced a significant increase in the expression of the apoptotic marker p53. This supports our recent results where we showed that WPS caused apoptosis in the hearts by increasing the levels of multiple apoptotic markers (Nemmar et al., 2023). Furthermore, *in vitro* findings showed that nicotine induces cardiomyocyte apoptosis by triggering oxidative stress and up-regulating the expression of genes associated with apoptosis such as p53 and Bax (Zhou et al., 2010). Our data show that Occ-WPS induced a non-significant elevation and Reg-WPS caused a significant augmentation in the cardiac expression of Nrf2. We have previously shown that WPS exposure induced an increase in the expression of cardiac Nrf2 (Nemmar et al., 2019; Nemmar et al., 2020; Nemmar et al., 2022). The increase of the Nrf2 expression was linked to the occurrence of oxidative stress and the initiation of an antioxidant response (Vargas-Mendoza et al., 2019).

Our data also showed that Reg-WPS exposure caused an alteration in the levels of mitochondrial complexes II & III, and IV activities. Moreover, Occ-WPS failed to increase significantly the activity of the complexes I, II & III and IV. Our results are in agreement with the study that revealed pleomorphic mitochondria with deteriorated and partially disrupted or disappeared cristae in the ventricular cardiomyocytes of WPS-exposed rats (Al-Awaida et al., 2015). Likewise, CS exposure for 3 months on a daily basis caused dysfunction and structural aberration of the mitochondria (Shehadeh, 2014).

Cotinine is the predominant metabolite of nicotine that is usually utilized as a biochemical marker for tobacco smoke exposure (Mattes et al., 2014). Our results revealed a significant increase of cotinine in the plasma of mice exposed to Reg-WPS compared with Occ-WPS and air-exposed groups. In Occ-WPS group cotinine was insignificantly increased compared with control.

It has been reported that whole-body exposure to WPS induces oxidative stress in cardiac tissue of adult male offspring rats (Al-Sawalha et al., 2019) and increases the risk of thrombosis in mice (Alarabi et al., 2020). Our data were obtained in a nose-only exposure system, and hence it would be interesting to verify whether the observed effect would be similar in whole-body exposure setup. Additional work is needed to address this point. This study has limitations, including the lack of the determination of the toxicants responsible for the observed effects including flavor-specific toxicity, studying various time points and the assessment of other biochemical and physiological cardiorespiratory outcomes in the same exposed animals. Moreover, in the current study, we did not provide evidence for mitochondrial dysfunction by showing the occurrence of fission/fusion, and dysregulation of associated proteins such as mitochondrial fusion promoter 1/2, dynamin-related protein, dominant optic atrophy 1, or mitochondrial membrane potential assessment. Further work is required to assess these points.

In conclusion, the current study demonstrated that regular exposure to WPS has a deleterious effect on the heart and its functions through the induction of cardiac damage, inflammation, and oxidative stress markers. Furthermore, WPS induced genotoxicity, mitochondrial dysfunction and cotinine in the plasma. Moreover, Occ-WPS exposure showed a trendy increase in almost all the measured parameters in addition to a significant effect on blood pressure, ANP, antioxidant enzymes, IL17, the chemokine CXCL1 and cardiac DNA damage.

## Data Availability

The original contributions presented in the study are included in the article/Supplementary Material, further inquiries can be directed to the corresponding author.
