# Supplementary material for: Impact of prolonged exposure to occasional and regular waterpipe smoke on cardiac injury, oxidative stress and mitochondrial dysfunction in male mice
Source: Front Physiol. 2024 Feb 2;15:1286366. doi: 10.3389/fphys.2024.1286366 (PMC10869456; doi:10.3389/fphys.2024.1286366)
Supplement: Supplementary file 1 [file DataSheet1.PDF]

Supplementary material

Statistical analysis (reanalysis of data as requested by reviewer #1)

We have reanalyzed all our data and compared the mean of each column with the mean of every other column using one-way analysis of variance ANOVA, followed by Holm-Sidak's multiple comparisons test. Thus, we have taken in consideration comparison air *vs* Occ-WPS, air *vs* Reg- WPS, and Occ-WPS *vs* Reg-WPS for all the assessed parameters.

Systolic blood pressure (SBP)

ANOVA results | Multiple comparisons

Ordinary one-way ANOVA
ANOVA results

1	Table Analyzed	SBP
2	Data sets analyzed	A-C
3		
4	**ANOVA summary**	
5	F	22.41
6	P value	<0.0001
7	P value summary	****
8	Significant diff. among means (P < 0.05)?	Yes
9	R squared	0.7888

ANOVA results | Multiple comparisons

Ordinary one-way ANOVA
Multiple comparisons

1	Number of families	1							
2	Number of comparisons per family	3							
3	Alpha	0.05							
4									
5	**Holm-Šidák's multiple comparisons test**	**Mean Diff.**	**Below threshold?**	**Summary**	**Adjusted P Value**				
6	Air vs. Occ-WPS	-23.60	Yes	**	0.0087	A-B			
7	Air vs. Reg-WPS	-49.54	Yes	****	<0.0001	A-C			
8	Occ-WPS vs. Reg-WPS	-25.94	Yes	**	0.0087	B-C			
9									
10	**Test details**	**Mean 1**	**Mean 2**	**Mean Diff.**	**SE of diff.**	**n1**	**n2**	**t**	**DF**
11	Air vs. Occ-WPS	95.72	119.3	-23.60	7.403	5	5	3.188	12
12	Air vs. Reg-WPS	95.72	145.3	-49.54	7.403	5	5	6.692	12
13	Occ-WPS vs. Reg-WPS	119.3	145.3	-25.94	7.403	5	5	3.504	12
14									

Troponin I

	Ordinary one-way ANOVA ANOVA results	
1	Table Analyzed	Non-parametric-Troponin I Heart
2	Data sets analyzed	A-C
3		
4	**ANOVA summary**	
5	F	11.20
6	P value	0.0005
7	P value summary	***
8	Significant diff. among means (P < 0.05)?	Yes
9	R squared	0.5161
10		

	Ordinary one-way ANOVA Multiple comparisons								
1	Number of families	1							
2	Number of comparisons per family	3							
3	Alpha	0.05							
4									
5	**Holm-Šidák's multiple comparisons test**	**Mean Diff.**	**Below threshold?**	**Summary**	**Adjusted P Value**				
6	Air vs. Occ-WPS	-147.6	No	ns	0.3237	A-B			
7	Air vs. Reg-WPS	-658.7	Yes	***	0.0006	A-C			
8	Occ-WPS vs. Reg-WPS	-511.1	Yes	**	0.0043	B-C			
9									
10	**Test details**	**Mean 1**	**Mean 2**	**Mean Diff.**	**SE of diff.**	**n1**	**n2**	**t**	**DF**
11	Air vs. Occ-WPS	245.3	392.9	-147.6	146.1	8	8	1.011	21
12	Air vs. Reg-WPS	245.3	904.0	-658.7	146.1	8	8	4.509	21
13	Occ-WPS vs. Reg-WPS	392.9	904.0	-511.1	146.1	8	8	3.499	21
14									

Brain natriuretic peptide (BNP)

ANOVA results | Multiple comparisons

Ordinary one-way ANOVA
ANOVA results

1	Table Analyzed	BNP Heart
2	Data sets analyzed	A-C
3		
4	**ANOVA summary**	
5	F	16.11
6	P value	<0.0001
7	P value summary	****
8	Significant diff. among means (P < 0.05	Yes
9	R squared	0.6055

ANOVA results | Multiple comparisons

Ordinary one-way ANOVA
Multiple comparisons

1	Number of families	1							
2	Number of comparisons per family	3							
3	Alpha	0.05							
4									
5	**Holm-Šidák's multiple comparisons test**	**Mean Diff.**	**Below threshold?**	**Summary**	**Adjusted P Value**				
6	Air vs. Occ-WPS	-105.4	No	ns	0.1521	A-B			
7	Air vs. Reg-WPS	-389.1	Yes	****	<0.0001	A-C			
8	Occ-WPS vs. Reg-WPS	-283.7	Yes	**	0.0013	B-C			
9									
10	**Test details**	**Mean 1**	**Mean 2**	**Mean Diff.**	**SE of diff.**	**n1**	**n2**	**t**	**DF**
11	Air vs. Occ-WPS	17.58	122.9	-105.4	70.89	8	8	1.486	21
12	Air vs. Reg-WPS	17.58	406.6	-389.1	70.89	8	8	5.488	21
13	Occ-WPS vs. Reg-WPS	122.9	406.6	-283.7	70.89	8	8	4.002	21
14									

Atrial natriuretic peptide (ANP)

1	Table Analyzed	ANP Heart
2	Data sets analyzed	A-C
3		
4	**ANOVA summary**	
5	F	27.09
6	P value	<0.0001
7	P value summary	****
8	Significant diff. among means (P < 0.05	Yes
9	R squared	0.7206

	Ordinary one-way ANOVA<br>Multiple comparisons								
1	Number of families	1							
2	Number of comparisons per family	3							
3	Alpha	0.05							
4									
5	**Holm-Šidák's multiple comparisons test**	**Mean Diff.**	**Below threshold?**	**Summary**	**Adjusted P Value**				
6	Air vs. Occ-WPS	-3.100	Yes	***	0.0003	A-B			
7	Air vs. Reg-WPS	-4.800	Yes	****	<0.0001	A-C			
8	Occ-WPS vs. Reg-WPS	-1.700	Yes	*	0.0178	B-C			
9									
10	**Test details**	**Mean 1**	**Mean 2**	**Mean Diff.**	**SE of diff.**	**n1**	**n2**	**t**	**DF**
11	Air vs. Occ-WPS	9.400	12.50	-3.100	0.6613	8	8	4.687	21
12	Air vs. Reg-WPS	9.400	14.20	-4.800	0.6613	8	8	7.258	21
13	Occ-WPS vs. Reg-WPS	12.50	14.20	-1.700	0.6613	8	8	2.571	21
14									
15									

Lactate dehydrogenase (LDH)

	Ordinary one-way ANOVA<br>ANOVA results	
1	Table Analyzed	Non-parametric-LDH Heart
2	Data sets analyzed	A-C
3		
4	**ANOVA summary**	
5	F	8.368
6	P value	0.0021
7	P value summary	**
8	Significant diff. among means (P < 0.05)?	Yes
9	R squared	0.4435

	Ordinary one-way ANOVA<br>Multiple comparisons								
1	Number of families	1							
2	Number of comparisons per family	3							
3	Alpha	0.05							
4									
5	**Holm-Šidák's multiple comparisons test**	**Mean Diff.**	**Below threshold?**	**Summary**	**Adjusted P Value**				
6	Air vs. Occ-WPS	-0.4375	No	ns	0.4535	A-B			
7	Air vs. Reg-WPS	-2.213	Yes	**	0.0027	A-C			
8	Occ-WPS vs. Reg-WPS	-1.775	Yes	*	0.0109	B-C			
9									
10	**Test details**	**Mean 1**	**Mean 2**	**Mean Diff.**	**SE of diff.**	**n1**	**n2**	**t**	**DF**
11	Air vs. Occ-WPS	1.488	1.925	-0.4375	0.5728	8	8	0.7637	21
12	Air vs. Reg-WPS	1.488	3.700	-2.213	0.5728	8	8	3.862	21
13	Occ-WPS vs. Reg-WPS	1.925	3.700	-1.775	0.5728	8	8	3.099	21

Creatine phosphokinase (CK)

	Ordinary one-way ANOVA<br>ANOVA results	
1	Table Analyzed	CK Heart
2	Data sets analyzed	A-C
3		
4	**ANOVA summary**	
5	F	18.87
6	P value	<0.0001
7	P value summary	****
8	Significant diff. among means (P < 0.05)?	Yes
9	R squared	0.6425

	Ordinary one-way ANOVA<br>Multiple comparisons								
1	Number of families	1							
2	Number of comparisons per family	3							
3	Alpha	0.05							
4									
5	**Holm-Šídák's multiple comparisons test**	**Mean Diff.**	**Below threshold?**	**Summary**	**Adjusted P Value**				
6	Air vs. Occ-WPS	-0.3750	No	ns	0.5695	A-B			
7	Air vs. Reg-WPS	-3.625	Yes	****	<0.0001	A-C			
8	Occ-WPS vs. Reg-WPS	-3.250	Yes	***	0.0001	B-C			
9									
10	**Test details**	**Mean 1**	**Mean 2**	**Mean Diff.**	**SE of diff.**	**n1**	**n2**	**t**	**DF**
11	Air vs. Occ-WPS	2.375	2.750	-0.3750	0.6489	8	8	0.5779	21
12	Air vs. Reg-WPS	2.375	6.000	-3.625	0.6489	8	8	5.586	21
13	Occ-WPS vs. Reg-WPS	2.750	6.000	-3.250	0.6489	8	8	5.008	21

Lipid peroxidation (LPO)

Ordinary one-way ANOVA<br>ANOVA results	
Table Analyzed	LPO Heart
Data sets analyzed	A-C
**ANOVA summary**	
F	3.626
P value	0.0453
P value summary	*
Significant diff. among means (P < 0.05	Yes
R squared	0.2661

Ordinary one-way ANOVA<br>Multiple comparisons								
Number of families	1							
Number of comparisons per family	3							
Alpha	0.05							
**Holm-Šidák's multiple comparisons test**	**Mean Diff.**	**Below threshold?**	**Summary**	**Adjusted P Value**				
Air vs. Occ-WPS	-1.296	No	ns	0.2590	A-B			
Air vs. Reg-WPS	-2.263	Yes	*	0.0416	A-C			
Occ-WPS vs. Reg-WPS	-0.9675	No	ns	0.2590	B-C			
**Test details**	**Mean 1**	**Mean 2**	**Mean Diff.**	**SE of diff.**	**n1**	**n2**	**t**	**DF**
Air vs. Occ-WPS	2.514	3.810	-1.296	0.8413	7	8	1.540	20
Air vs. Reg-WPS	2.514	4.778	-2.263	0.8413	7	8	2.690	20
Occ-WPS vs. Reg-WPS	3.810	4.778	-0.9675	0.8128	8	8	1.190	20

Reduced glutathione (GSH)

Ordinary one-way ANOVA<br>ANOVA results	
Table Analyzed	GSH Heart
Data sets analyzed	A-C
**ANOVA summary**	
F	5.871
P value	0.0109
P value summary	*
Significant diff. among means (P < 0.05)?	Yes
R squared	0.3948

Ordinary one-way ANOVA<br>Multiple comparisons								
Number of families	1							
Number of comparisons per family	3							
Alpha	0.05							
**Holm-Šidák's multiple comparisons test**	**Mean Diff.**	**Below threshold?**	**Summary**	**Adjusted P Value**				
Air vs. Occ-WPS	-1.399	No	ns	0.1672	A-B			
Air vs. Reg-WPS	-2.961	Yes	**	0.0090	A-C			
Occ-WPS vs. Reg-WPS	-1.563	No	ns	0.1672	B-C			
**Test details**	**Mean 1**	**Mean 2**	**Mean Diff.**	**SE of diff.**	**n1**	**n2**	**t**	**DF**
Air vs. Occ-WPS	3.893	5.291	-1.399	0.8647	7	7	1.617	18
Air vs. Reg-WPS	3.893	6.854	-2.961	0.8647	7	7	3.425	18
Occ-WPS vs. Reg-WPS	5.291	6.854	-1.563	0.8647	7	7	1.807	18

Catalase

Table Analyzed	CAT Heart
Data sets analyzed	A-C
**ANOVA summary**	
F	47.15
P value	<0.0001
P value summary	****
Significant diff. among means (P < 0.05)?	Yes
R squared	0.8179

Number of families	1							
Number of comparisons per family	3							
Alpha	0.05							
**Holm-Šidák's multiple comparisons test**	**Mean Diff.**	**Below threshold?**	**Summary**	**Adjusted P Value**				
Air vs. Occ-WPS	-36.13	Yes	*	0.0315	A-B			
Air vs. Reg-WPS	-146.1	Yes	****	<0.0001	A-C			
Occ-WPS vs. Reg-WPS	-110.0	Yes	****	<0.0001	B-C			
**Test details**	**Mean 1**	**Mean 2**	**Mean Diff.**	**SE of diff.**	**n1**	**n2**	**t**	**DF**
Air vs. Occ-WPS	102.5	138.6	-36.13	15.68	8	8	2.305	21
Air vs. Reg-WPS	102.5	248.6	-146.1	15.68	8	8	9.322	21
Occ-WPS vs. Reg-WPS	138.6	248.6	-110.0	15.68	8	8	7.017	21

Superoxide dismutase (SOD)

Table Analyzed	SOD Heart
Data sets analyzed	A-C
ANOVA summary	
F	37.04
P value	<0.0001
P value summary	****
Significant diff. among means (P < 0.05)?	Yes
R squared	0.7791

Number of families	1							
Number of comparisons per family	3							
Alpha	0.05							
Holm-Šidák's multiple comparisons test	Mean Diff.	Below threshold?	Summary	Adjusted P Value				
Air vs. Occ-WPS	-21.13	Yes	***	0.0004	A-B			
Air vs. Reg-WPS	-43.71	Yes	****	<0.0001	A-C			
Occ-WPS vs. Reg-WPS	-22.59	Yes	***	0.0004	B-C			
Test details	Mean 1	Mean 2	Mean Diff.	SE of diff.	n1	n2	t	DF
Air vs. Occ-WPS	34.16	55.29	-21.13	5.080	8	8	4.159	21
Air vs. Reg-WPS	34.16	77.88	-43.71	5.080	8	8	8.605	21
Occ-WPS vs. Reg-WPS	55.29	77.88	-22.59	5.080	8	8	4.446	21

Monocyte chemoattractant protein-1 (MCP-1)

Table Analyzed	MCP-1
Data sets analyzed	A-C
**ANOVA summary**	
F	12.40
P value	0.0003
P value summary	***
Significant diff. among means (P < 0.05	Yes
R squared	0.5415

Number of families	1							
Number of comparisons per family	3							
Alpha	0.05							
**Holm-Šidák's multiple comparisons test**	**Mean Diff.**	**Below threshold?**	**Summary**	**Adjusted P Value**				
Air vs. Occ-WPS	-0.3813	No	ns	0.0639	A-B			
Air vs. Reg-WPS	-0.9638	Yes	***	0.0002	A-C			
Occ-WPS vs. Reg-WPS	-0.5825	Yes	*	0.0140	B-C			
**Test details**	**Mean 1**	**Mean 2**	**Mean Diff.**	**SE of diff.**	**n1**	**n2**	**t**	**DF**
Air vs. Occ-WPS	0.2050	0.5863	-0.3813	0.1949	8	8	1.956	21
Air vs. Reg-WPS	0.2050	1.169	-0.9638	0.1949	8	8	4.944	21
Occ-WPS vs. Reg-WPS	0.5863	1.169	-0.5825	0.1949	8	8	2.988	21

Chemokine CXCL1

Table Analyzed	CXCL1
Data sets analyzed	A-C
ANOVA summary	
F	43.27
P value	<0.0001
P value summary	****
Significant diff. among means (P < 0.05	Yes
R squared	0.8047

Number of families	1				
Number of comparisons per family	3				
Alpha	0.05				
Holm-Šidák's multiple comparisons test	Mean Diff.	Below threshold?	Summary	Adjusted P Value	
Air vs. Occ-WPS	-61.60	Yes	****	<0.0001	A-B
Air vs. Reg-WPS	-109.9	Yes	****	<0.0001	A-C
Occ-WPS vs. Reg-WPS	-48.33	Yes	***	0.0005	B-C

Test details	Mean 1	Mean 2	Mean Diff.	SE of diff.	n1	n2	t	DF
Air vs. Occ-WPS	35.80	97.40	-61.60	11.85	8	8	5.200	21
Air vs. Reg-WPS	35.80	145.7	-109.9	11.85	8	8	9.280	21
Occ-WPS vs. Reg-WPS	97.40	145.7	-48.33	11.85	8	8	4.080	21

E-selectin

Table Analyzed	E-Selectin
Data sets analyzed	A-C
**ANOVA summary**	
F	43.20
P value	<0.0001
P value summary	****
Significant diff. among means (P < 0.05	Yes
R squared	0.8120

Number of families	1
Number of comparisons per family	3
Alpha	0.05

Holm-Šidák's multiple comparisons test	Mean Diff.	Below threshold?	Summary	Adjusted P Value	
Air vs. Occ-WPS	-18.66	No	ns	0.1522	A-B
Air vs. Reg-WPS	-113.6	Yes	****	<0.0001	A-C
Occ-WPS vs. Reg-WPS	-94.90	Yes	****	<0.0001	B-C

Test details	Mean 1	Mean 2	Mean Diff.	SE of diff.	n1	n2	t	DF
Air vs. Occ-WPS	36.79	55.45	-18.66	12.53	8	8	1.489	20
Air vs. Reg-WPS	36.79	150.4	-113.6	12.97	8	7	8.753	20
Occ-WPS vs. Reg-WPS	55.45	150.4	-94.90	12.97	8	7	7.315	20

Vascular cell adhesion molecule-1 (VCAM-1)

Table Analyzed	VCAM-1
Data sets analyzed	A-C
**ANOVA summary**	
F	4.848
P value	0.0186
P value summary	*
Significant diff. among means (P < 0.05)	Yes
R squared	0.3159

Number of families	1							
Number of comparisons per family	3							
Alpha	0.05							
**Holm-Šídák's multiple comparisons test**	**Mean Diff.**	**Below threshold?**	**Summary**	**Adjusted P Value**				
Air vs. Occ-WPS	-1.500	No	ns	0.2208	A-B			
Air vs. Reg-WPS	-3.680	Yes	*	0.0163	A-C			
Occ-WPS vs. Reg-WPS	-2.180	No	ns	0.1551	B-C			
**Test details**	**Mean 1**	**Mean 2**	**Mean Diff.**	**SE of diff.**	**n1**	**n2**	**t**	**DF**
Air vs. Occ-WPS	5.196	6.696	-1.500	1.189	8	8	1.262	21
Air vs. Reg-WPS	5.196	8.876	-3.680	1.189	8	8	3.096	21
Occ-WPS vs. Reg-WPS	6.696	8.876	-2.180	1.189	8	8	1.834	21

Intercellular adhesion molecule-1 (ICAM-1)

Table Analyzed	ICAM-1
Data sets analyzed	A-C
**ANOVA summary**	
F	3 433
P value	0 0523
P value summary	ns
Significant diff. among means (P < 0 05	No
R squared	0 2556

Number of families	1								
Number of comparisons per family	3								
Alpha	0.05								
**Holm-Šídák's multiple comparisons test**	**Mean Diff.**	**Below threshold?**	**Summary**	**Adjusted P Value**					
Air vs. Occ-WPS	-2.384	No	ns	0.3709	A-B				
Air vs. Reg-WPS	-6.987	No	ns	0.0514	A-C				
Occ-WPS vs. Reg-WPS	-4.603	No	ns	0.1957	B-C				
**Test details**	**Mean 1**	**Mean 2**	**Mean Diff.**	**SE of diff.**	**n1**	**n2**	**t**	**DF**	
Air vs. Occ-WPS	8.160	10.54	-2.384	2.604	8	8	0.9154	20	
Air vs. Reg-WPS	8.160	15.15	-6.987	2.696	8	7	2.592	20	
Occ-WPS vs. Reg-WPS	10.54	15.15	-4.603	2.696	8	7	1.708	20	

Interleukin-1β (IL-1β)

Table Analyzed	IL1B				
Data sets analyzed	A-C				
**ANOVA summary**					
F	5.947				
P value	0.0094				
P value summary	**				
Significant diff. among means (P < 0.05	Yes				
R squared	0.3729				

Number of families	1							
Number of comparisons per family	3							
Alpha	0.05							
**Holm-Šidák's multiple comparisons test**	**Mean Diff.**	**Below threshold?**	**Summary**	**Adjusted P Value**				
Air vs. Occ-WPS	-117.3	No	ns	0.1703	A-B			
Air vs. Reg-WPS	-235.1	Yes	**	0.0076	A-C			
Occ-WPS vs. Reg-WPS	-117.8	No	ns	0.1703	B-C			
**Test details**	**Mean 1**	**Mean 2**	**Mean Diff.**	**SE of diff.**	**n1**	**n2**	**t**	**DF**
Air vs. Occ-WPS	88.22	205.5	-117.3	68.22	7	8	1.719	20
Air vs. Reg-WPS	88.22	323.3	-235.1	68.22	7	8	3.446	20
Occ-WPS vs. Reg-WPS	205.5	323.3	-117.8	65.91	8	8	1.787	20

Tumor necrosis factor α (TNFα)

Table Analyzed	TNF
Data sets analyzed	A-C
**ANOVA summary**	
F	11.27
P value	0.0006
P value summary	***
Significant diff. among means (P < 0.05)?	Yes
R squared	0.5426

Number of families	1							
Number of comparisons per family	3							
Alpha	0.05							
**Holm-Šídák's multiple comparisons test**	**Mean Diff.**	**Below threshold?**	**Summary**	**Adjusted P Value**				
Air vs. Occ-WPS	45.15	No	ns	0.4895	A-B			
Air vs. Reg-WPS	-221.0	Yes	**	0.0054	A-C			
Occ-WPS vs. Reg-WPS	-266.2	Yes	***	0.0008	B-C			
**Test details**	**Mean 1**	**Mean 2**	**Mean Diff.**	**SE of diff.**	**n1**	**n2**	**t**	**DF**
Air vs. Occ-WPS	359.4	314.3	45.15	64.06	6	8	0.7048	19
Air vs. Reg-WPS	359.4	580.5	-221.0	64.06	6	8	3.450	19
Occ-WPS vs. Reg-WPS	314.3	580.5	-266.2	59.31	8	8	4.488	19

Interleukin 17 (IL17)

Table Analyzed	IL17
Data sets analyzed	A-C
**ANOVA summary**	
F	90.37
P value	<0.0001
P value summary	****
Significant diff. among means (P < 0.05)?	Yes
R squared	0.8959

Number of families	1							
Number of comparisons per family	3							
Alpha	0.05							
**Holm-Šidák's multiple comparisons test**	**Mean Diff.**	**Below threshold?**	**Summary**	**Adjusted P Value**				
Air vs. Occ-WPS	-44.01	Yes	****	<0.0001	A-B			
Air vs. Reg-WPS	-77.91	Yes	****	<0.0001	A-C			
Occ-WPS vs. Reg-WPS	-33.90	Yes	****	<0.0001	B-C			
**Test details**	**Mean 1**	**Mean 2**	**Mean Diff.**	**SE of diff.**	**n1**	**n2**	**t**	**DF**
Air vs. Occ-WPS	25.94	69.95	-44.01	5.812	8	8	7.573	21
Air vs. Reg-WPS	25.94	103.9	-77.91	5.812	8	8	13.41	21
Occ-WPS vs. Reg-WPS	69.95	103.9	-33.90	5.812	8	8	5.833	21

DNA migration

Table Analyzed	DNA damage				
Data sets analyzed	A-C				
ANOVA summary					
F	512.1				
P value	<0.0001				
P value summary	****				
Significant diff. among means (P < 0.05	Yes				
R squared	0.9884				

Number of families	1							
Number of comparisons per family	3							
Alpha	0.05							
Holm-Šidák's multiple comparisons test	Mean Diff.	Below threshold?	Summary	Adjusted P Value				
Air vs. Occ-WPS	-2.532	Yes	**	0.0013	A-B			
Air vs. Reg-WPS	-17.98	Yes	****	<0.0001	A-C			
Occ-WPS vs. Reg-WPS	-15.44	Yes	****	<0.0001	B-C			
Test details	Mean 1	Mean 2	Mean Diff.	SE of diff.	n1	n2	t	DF
Air vs. Occ-WPS	9.424	11.96	-2.532	0.6081	5	5	4.164	12
Air vs. Reg-WPS	9.424	27.40	-17.98	0.6081	5	5	29.56	12
Occ-WPS vs. Reg-WPS	11.96	27.40	-15.44	0.6081	5	5	25.40	12

Mammalian target of rapamycin (mTOR)

Table Analyzed	mTOR
Data sets analyzed	A-C
**ANOVA summary**	
F	5.616
P value	0.0111
P value summary	*
Significant diff. among means (P < 0.05	Yes
R squared	0.3485

Number of families	1							
Number of comparisons per family	3							
Alpha	0.05							
**Holm-Šidák's multiple comparisons test**	**Mean Diff.**	**Below threshold?**	**Summary**	**Adjusted P Value**				
Air vs. Occ-WPS	-0.04325	No	ns	0.2550	A-B			
Air vs. Reg-WPS	-0.1221	Yes	*	0.0101	A-C			
Occ-WPS vs. Reg-WPS	-0.07888	No	ns	0.0875	B-C			
**Test details**	**Mean 1**	**Mean 2**	**Mean Diff.**	**SE of diff.**	**n1**	**n2**	**t**	**DF**
Air vs. Occ-WPS	0.08363	0.1269	-0.04325	0.03695	8	8	1.170	21
Air vs. Reg-WPS	0.08363	0.2058	-0.1221	0.03695	8	8	3.305	21
Occ-WPS vs. Reg-WPS	0.1269	0.2058	-0.07888	0.03695	8	8	2.134	21

p53

Table Analyzed	P53
Data sets analyzed	A-C
**ANOVA summary**	
F	14.05
P value	0.0001
P value summary	***
Significant diff. among means (P < 0.05)?	Yes
R squared	0.5723

Number of families	1							
Number of comparisons per family	3							
Alpha	0.05							
**Holm-Šídák's multiple comparisons test**	**Mean Diff.**	**Below threshold?**	**Summary**	**Adjusted P Value**				
Air vs. Occ-WPS	0.2625	No	ns	0.6160	A-B			
Air vs. Reg-WPS	-2.225	Yes	***	0.0006	A-C			
Occ-WPS vs. Reg-WPS	-2.488	Yes	***	0.0003	B-C			
**Test details**	**Mean 1**	**Mean 2**	**Mean Diff.**	**SE of diff.**	**n1**	**n2**	**t**	**DF**
Air vs. Occ-WPS	1.838	1.575	0.2625	0.5156	8	8	0.5091	21
Air vs. Reg-WPS	1.838	4.063	-2.225	0.5156	8	8	4.315	21
Occ-WPS vs. Reg-WPS	1.575	4.063	-2.488	0.5156	8	8	4.824	21

Nuclear factor erythroid-derived 2-like 2 (Nrf2)

Table Analyzed	NRF2-IHC
Data sets analyzed	A-C
**ANOVA summary**	
F	27.66
P value	<0.0001
P value summary	****
Significant diff. among means (P < 0.05	Yes
R squared	0.7545

Number of families	1							
Number of comparisons per family	3							
Alpha	0.05							
**Holm-Šidák's multiple comparisons test**	**Mean Diff.**	**Below threshold?**	**Summary**	**Adjusted P Value**				
Air vs. Int-WPS	-9.457	No	ns	0.0633	A-B			
Air vs. Reg-WPS	-34.40	Yes	****	<0.0001	A-C			
Int-WPS vs. Reg-WPS	-24.94	Yes	***	0.0001	B-C			
**Test details**	**Mean 1**	**Mean 2**	**Mean Diff.**	**SE of diff.**	**n1**	**n2**	**t**	**DF**
Air vs. Int-WPS	19.59	29.04	-9.457	4.778	7	7	1.979	18
Air vs. Reg-WPS	19.59	53.99	-34.40	4.778	7	7	7.199	18
Int-WPS vs. Reg-WPS	29.04	53.99	-24.94	4.778	7	7	5.220	18

Mitochondrial complex I

Table Analyzed	Mitochondrial Complex I
Data sets analyzed	A-C
**ANOVA summary**	
F	3.666
P value	0.0505
P value summary	ns
Significant diff. among means (P < 0.0	No
R squared	0.3283

Number of families	1								
Number of comparisons per family	3								
Alpha	0.05								
Holm-Šídák's multiple comparisons test	Mean Diff.	Below threshold?	Summary	Adjusted P Value					
Air vs. Occ-WPS	-0.01983	No	ns	0.7334	A-B				
Air vs. Reg-WPS	-0.1428	No	ns	0.0718	A-C				
Occ-WPS vs. Reg-WPS	-0.1230	No	ns	0.0938	B-C				
Test details	Mean 1	Mean 2	Mean Diff.	SE of diff.	n1	n2	t	DF	
Air vs. Occ-WPS	0.08933	0.1092	-0.01983	0.05715	6	6	0.3470	15	
Air vs. Reg-WPS	0.08933	0.2322	-0.1428	0.05715	6	6	2.499	15	
Occ-WPS vs. Reg-WPS	0.1092	0.2322	-0.1230	0.05715	6	6	2.152	15	

Mitochondrial complexes II & III

Table Analyzed	Mitochondrial Complex II and III
Data sets analyzed	A-C
**ANOVA summary**	
F	25.28
P value	<0.0001
P value summary	****
Significant diff. among means (P < 0.0:	Yes
R squared	0.7712

Number of families	1							
Number of comparisons per family	3							
Alpha	0.05							
Holm-Šídák's multiple comparisons test	Mean Diff.	Below threshold?	Summary	Adjusted P Value				
Air vs. Occ-WPS	-0.01383	No	ns	0.2495	A-B			
Air vs. Reg-WPS	-0.07700	Yes	****	<0.0001	A-C			
Occ-WPS vs. Reg-WPS	-0.06317	Yes	***	0.0001	B-C			
Test details	Mean 1	Mean 2	Mean Diff.	SE of diff.	n1	n2	t	DF
Air vs. Occ-WPS	0.07500	0.08883	-0.01383	0.01155	6	6	1.198	15
Air vs. Reg-WPS	0.07500	0.1520	-0.07700	0.01155	6	6	6.669	15
Occ-WPS vs. Reg-WPS	0.08883	0.1520	-0.06317	0.01155	6	6	5.471	15

Mitochondrial complex IV

Table Analyzed	Mitochondrial Complex IV
Data sets analyzed	A-C
**ANOVA summary**	
F	9.556
P value	0.0021
P value summary	**
Significant diff. among means (P < 0.0	Yes
R squared	0.5603

Number of families	1							
Number of comparisons per family	3							
Alpha	0.05							
**Holm-Šidák's multiple comparisons test**	**Mean Diff.**	**Below threshold?**	**Summary**	**Adjusted P Value**				
Air vs. Occ-WPS	-7.500e-005	No	ns	0.8278	A-B			
Air vs. Reg-WPS	-0.001318	Yes	**	0.0043	A-C			
Occ-WPS vs. Reg-WPS	-0.001243	Yes	**	0.0045	B-C			
**Test details**	**Mean 1**	**Mean 2**	**Mean Diff.**	**SE of diff.**	**n1**	**n2**	**t**	**DF**
Air vs. Occ-WPS	0.001802	0.001877	-7.500e-005	0.0003387	6	6	0.2214	15
Air vs. Reg-WPS	0.001802	0.003120	-0.001318	0.0003387	6	6	3.892	15
Occ-WPS vs. Reg-WPS	0.001877	0.003120	-0.001243	0.0003387	6	6	3.670	15

Immunohistochemistry negative control

(as requested by reviewer #1)

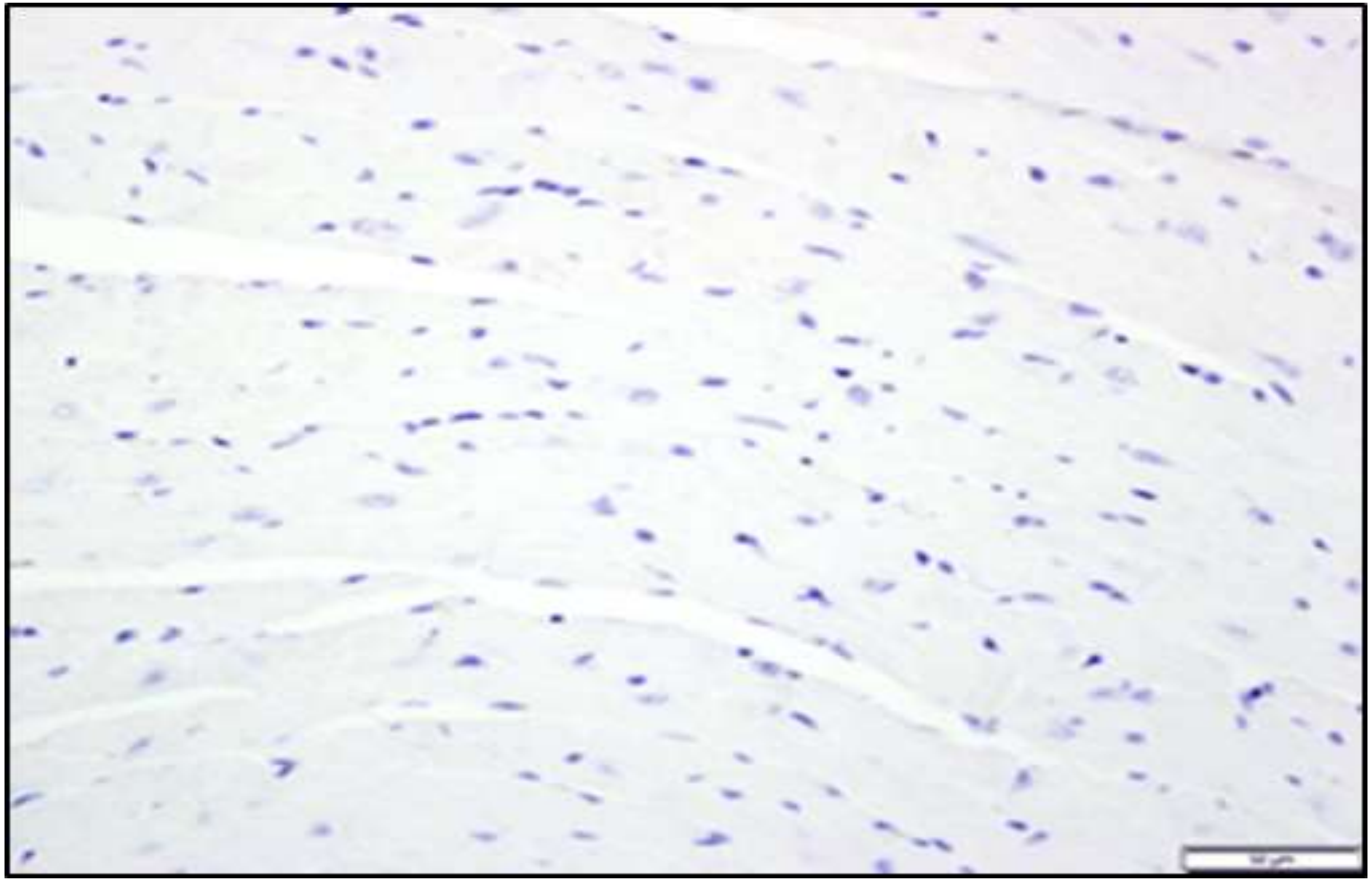

Supplementary Figure 1: Negative control of immunohistochemical staining of the heart tissue sections of mice for the detection of nuclear factor erythroid-derived 2-like 2 (Nrf2). The primary antibody for the detection of Nrf2 was not added to the heart sections. Scale bars: 50µm.
